# Wireless, Programmable, and Refillable Hydrogel Bioelectronics for Enhanced Diabetic Wound Healing

**DOI:** 10.1002/advs.202407820

**Published:** 2024-10-14

**Authors:** Ningjie Du, Yunlong Fan, Yunting Zhang, Hao Huang, Yidan Lyu, Ruisi Cai, Yuqi Zhang, Tianyuan Zhang, Yixin Guan, Kewang Nan

**Affiliations:** ^1^ College of Chemical and Biological Engineering Zhejiang University 866 Yuhangtang Road Hangzhou 310058 China; ^2^ College of Pharmaceutical Sciences Zhejiang University 866 Yuhangtang Road Hangzhou 310058 China; ^3^ MicroTech Medical (Hangzhou) Co., Ltd. 108 Liuze Road Hangzhou Zhejiang 311100 China; ^4^ Jinhua Institute of Zhejiang University Jinhua 321002 China; ^5^ Department of Burns and Wound Care Center The Second Affiliated Hospital Zhejiang University School of Medicine Zhejiang University 866 Yuhangtang Road Hangzhou 310058 China; ^6^ Department of Gastroenterology Surgery The Second Affiliated Hospital Zhejiang University School of Medicine Zhejiang University 866 Yuhangtang Road Hangzhou 310058 China

**Keywords:** diabetic wound healing, drug delivery, electrostimulation, hydrogel bioelectronics, wireless wearable electronics

## Abstract

Diabetic wounds, characterized by complex pathogenesis and high infection rates, pose significant challenges in treatment due to prolonged recovery times and high recurrence rates, often leading to severe complications such as amputation and death. Traditional dry dressing treatments fail to address the unique microenvironment of diabetic wounds and tend to cause secondary damage due to frequent replacement. In this study, an electronic‐embedding, drug‐loading hydrogel bioelectronics is reported for accelerating diabetic wound healing using a combination of programmable pharmaceutical and electrostimulative approaches. Encapsulated in stretchable and biocompatible materials, this device is capable of multiple drug refilling and accelerated drug release modulated by on‐board electronics. In vivo experiments on diabetic model rats confirm the device's effectiveness in promoting wound healing. This innovative approach implies the potential for improving diabetic wound management using a combination of physical, material, and pharmaceutical interventions.

## Graphical abstract

This study reports an electronic‐embedded, drug‐loading hydrogel bioelectronics for accelerating diabetic wound healing. The wearable device combining programmable drug release and electrostimulative are shown to effectively accelerate the healing of diabetic wounds in rat models. Its refillable and remotely programmable features support versatile modalities for diabetic wound management, promoting the development of chronic, customizable diabetic wound treatment.

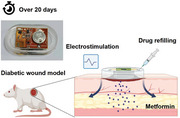

## 1 Introduction

Poor wound healing is one of the major complications of diabetes, affecting ≈20% of diabetic patients.^[^ ^1^ ^]^ Diabetic wounds are characterized by complex pathogenesis, high infection probability, long recovery time, and high recurrence rate. They increase risks of amputation and death, bringing heavy burden on patient's family and the entire healthcare system.^[^ ^2^, ^3^, ^4^ ^]^ The high blood glucose levels in diabetic patients can cause bacteria to grow faster than in normal wounds, and the immune system is less effective on killing bacteria at the wound site.^[^ ^5^ ^]^

Traditional treatment for diabetic wounds mainly involves covering the wound with dry dressings to absorb exudate and prevent infection. However, this approach does not address the specific microenvironment of diabetic wounds and may cause secondary damage due to adhesion between the dressing and the wound.^[^ ^6^ ^]^ Functional wound dressings such as hydrogels, nanofibers, hydrophilic colloids, and foams can provide a moist, antibacterial healing environment and carry bioactive agents or drugs to accelerate wound healing.^[^ ^7^ ^]^ Hydrogels, in particular, exhibit physical and chemical properties similar to natural extracellular matrices, and their 3D, porous structures facilitate drug loading, offering an ideal platform for wound management.^[^ ^8^, ^9^, ^10^ ^]^

However, existing hydrogel dressings have several shortcomings. First, due to the limited drug storage capacity and short duration, hydrogel dressings need frequent replacement, potentially causing secondary damage, as well as reduced patient adherence to medication.^[^ ^11^ ^]^ Second, most hydrogels rely on passive diffusion as the drug release mechanism, where the rate is heavily dependent on the drug concentration. As drug concentration gradually decreases over time, the effectiveness on the wound also diminishes accordingly.^[^ ^4^ ^]^ Furthermore, although some stimuli‐responsive drug‐eluting hydrogels have been reported,^[^ ^12^, ^13^ ^]^ the aforementioned limitations make controlled rhythmic drug delivery over a prolonged wound healing process challenging.

Electrostimulation is an effective method for accelerating wound healing.^[^ ^14^, ^15^, ^16^ ^]^ Many studies have combined it with soft electrodes, sometimes consisting of conductive hydrogels, to reduce the discomfort caused by rigid electrodes at the wound site.^[^ ^16^, ^17^, ^18^, ^19^ ^]^ Existing electrode technologies, however, lack programmability that takes into consideration the rhythmic changes in blood glucose levels that significantly impact wound healing, resulting in suboptimal therapeutic outcomes.^[^ ^20^ ^]^ Furthermore, the effectiveness of an integrated system combining pharmaceuticals, electrostimulation, and hydrogel dressing for effective diabetic wound management has not been investigated.

**Figure 1:**
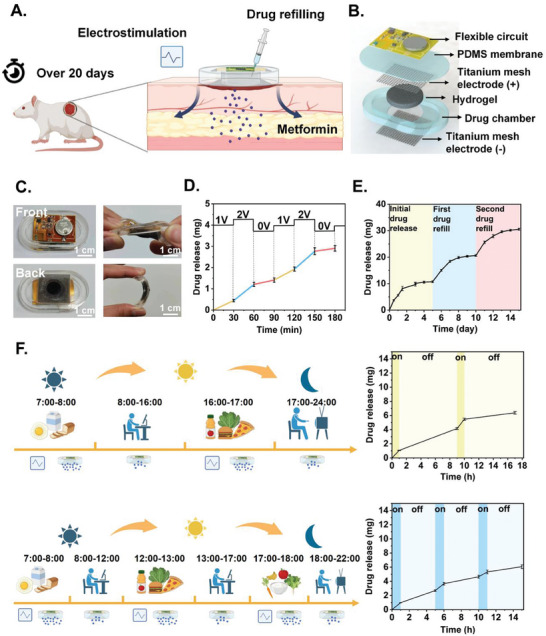
A) Functional schematic diagram of the hydrogel bioelectronics. The device features three main components: i) electrical controlled drug release hydrogel along with electrostimulation; ii) drug refillable chamber that can support continuous use for over 20 days; iii) programmable dosing rhythm under control of flexible circuit with Bluetooth. B) Exploded layered view of the device, including flexible circuit, PDMS membrane, and chamber, titanium mesh electrodes, and hydrogel. C) Appearance and mechanical properties of the device, which can maintain its functionality under torsional and bending forces. D) A drug release curve for cycles at 0, 1, and 2 V. The step curve represents the change of voltage on hydrogel controlled by MCU protocol on the device. The polyline represents the drug release rate. As the voltage increases, the slope of the drug release curve increases, indicating an enhanced drug release rate. E) Sustained drug release curves for multiple drug solution replenishments. Different colors indicate different times of drug refills. The drug release curve is relatively consistent after each drug refill. F) Specific drug release curves for the regular diet of diabetics. The line chart on the right corresponds to the simulated mealtime on the left. The drug release is accelerated during mealtime. The mealtime is simulated to be one hour. Data are shown as mean ± SD (standard deviation), *n* = 3.

Here, we report an electronic‐embedding, drug‐loading hydrogel bioelectronics that accelerates diabetic wound healing via a combination of programmable drug release and electrostimulation (**Figure** **1**A). In vitro characterizations of the hydrogel demonstrated its electrical conductivity, mechanical robustness, biocompatibility, and antibacterial properties. The device implemented a flexible, low‐power circuit with Bluetooth communication and voltage regulation functions, and programmable drug release profiles were verified through bench tests. In diabetic rodent models, the device remained functional for up to 21 days without change. Programmable electrostimulation and refillable hydrogel release systems together demonstrated accelerated wound healing in vivo. These and other results imply an integrated, long‐lasting, and effective electronic‐pharmaceutic hybrid solution for diabetic wound management.

## 2 Results and Discussion

Conventional hydrogel dressing for wound healing has limited drug‐loading capacity and requires frequent replacement, which leads to secondary damage and unstable drug release profiles. To address this, we designed a hydrogel bioelectronics where the drug can be refilled in situ without device replacement. Specifically, it consisted of three main components: conductive hydrogel, a soft chassis made of polydimethylsiloxane (PDMS), and flexible circuits (Figure 1B). The conductive hydrogel was molded into a cylindrical shape with a diameter of 20 mm and a thickness of 5 mm.

The soft chassis for drug refilling consisted of a chamber and a sealing membrane, both made of PDMS and assembled using instant adhesive pretreated with a primer that made it watertight (see Experimental Section for details). The chamber was designed to store up to 2 mL of drug solution that can be replenished with a regular syringe in situ. The refilled drug solution made consistent contact with the hydrogel to allow continuous diffusion of drug molecules into the hydrogel, thereby maintaining the drug concentration at a relatively constant level. A 20‐mm hole at the bottom of the chamber exposed the hydrogel to the wound sites.

A wireless flexible circuit was bonded on top of the soft chassis that comprised a coin cell battery, a microcontroller unit (MCU) with Bluetooth, a direct‐to‐direct current (DC‐DC) voltage regulator, and a digital potentiometer. Electrical contacts with the hydrogel were established using a pair of commercial titanium mesh electrodes (thickness = 0.1 mm, mesh count = 80). Although the chips and the battery were rigid, their small sizes and the flexible printed circuit board (flex‐PCB) underneath made of polyimide (thickness = 0.12 mm) ensured an overall flexibility of the device that did not interfere with wearing and regular body movements (Figure 1C).

The porous, 3D, and hydrophilic structure of the hydrogel permits the diffusion of substances into and out of the network. The addition of conductive fillers has been shown to influence the hydrogel network and electric charges, and led to controlled drug release through the hydrogel with voltage control.^[^ ^21^ ^]^ We explored and summarized various mechanisms for triggerable drug release from the hydrogel (Table S1, Supporting Information) and identified electrical stimulation as a suitable method for diabetic wound management. The hydrogel employed in this study consisted of chitosan (CS) and polyvinyl alcohol (PVA), with graphene oxide (GO) as the conductive filler and metformin as the model drug. CS, a natural polymer, has structural features resembling the extracellular matrix, making it suitable for cell growth, organization, and migration during tissue formation.^[^ ^22^ ^]^ PVA, commonly used in biomedical applications, is hydrophilic, non‐toxic, biocompatible, and exhibits good mechanical properties.^[^ ^23^ ^]^ The combination of chitosan and PVA in the hydrogel enhances its antibacterial, conductive, and swelling resistance properties.^[^ ^24^ ^]^ GO is chosen as the conductive filler due to its good hydrophilicity, dispersion, biocompatibility, and antibacterial properties, and mechanical strength.^[^ ^25^ ^]^

Metformin is a clinically approved drug for diabetic wound healing due to the superior performance in anti‐inflammation, anti‐senescence, and blood vessel protection.^[^ ^26^ ^]^ It accelerates the healing of diabetic wounds by activating the vascular endothelial growth factor (VEGF) pathway, which enhances angiogenesis in hyperglycemic conditions. It also mitigates hyperglycemia‐induced endothelial cell damage through mitophagy and inhibits the expression of pro‐inflammatory cytokines and adhesion molecules.^[^ ^27^ ^]^

As a proof‐of‐concept experiment, we wirelessly sent a mock DC voltage signal to the circuit, and observed a stepped change in drug release rate from the hydrogel that can be modulated by the voltage (Figure 1D), verifying our hypothesis that the drug release profiles can be tuned using wireless voltage control. At 0 V, the drug exhibited passive diffusion out of the hydrogel at a steady and slow rate, approximately half the rate observed at 1 V and a quarter of the rate at 2 V.

Refillability of the device was also demonstrated through a bench experiment. Over a 15‐days period, we found that a single refilling with 2 mL of 3% metformin solution allowed the hydrogel to keep releasing for up to 5 days (Figure 1E). 86.6% ± 2.0% of the total refilled drug were released during the 5‐days period, demonstrating a reasonable drug efficiency.

These and other capabilities of the hydrogel bioelectronics allowed for on‐demand drug release profiles according to personalized circadian rhythms. For example, patients with diabetes experience higher postprandial blood glucose peaks, which are more likely to induce oxidative stress and inflammatory responses at the wound sites.^[^ ^28^ ^]^ We demonstrated using bench experiments the capability of our device to adapt to such diabetic‐specific circadian rhythms. Specifically, the device can wirelessly switch between a dual‐pulse and a tri‐pulse release profile, each with clearly defined time intervals in <1 h temporal resolution, which adhered to mock dietary schedules representing two different patients (Figure 1F).

**Figure 2:**
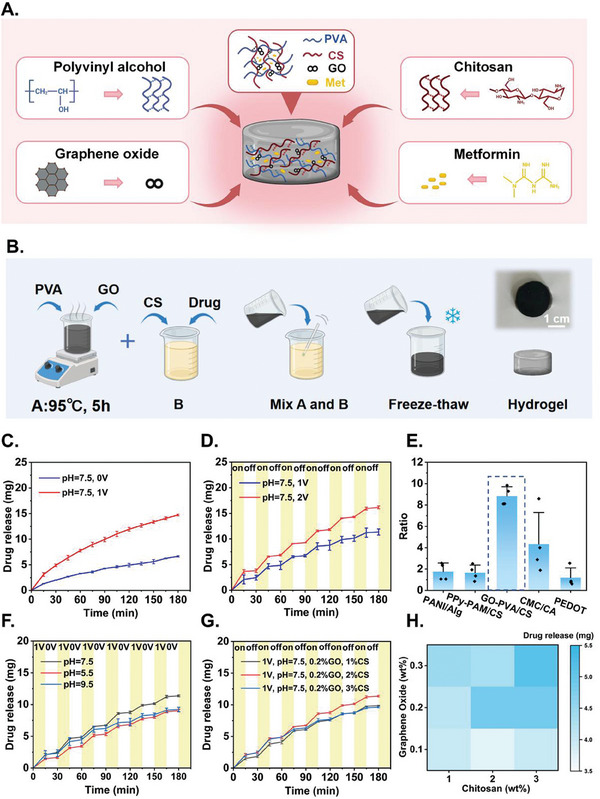
A) Composition and cross‐linked structure of the hydrogel. B) The synthesis steps of the hydrogel. C) Sustained drug release curves at 0 and 1 V over 180 min. D) Drug release curves at 1 and 2 V with 15 min interval. E) Release ratio of different hydrogels over 120‐min testing period. Error bars indicate the change of release ratio over time. F) Drug release curves at different pH with 15 min interval. G) Drug release curves with different CS content with 15 min interval. H) The synergistic effect of CS and GO content in drug release. Data are shown as mean ± SD, *n* = 3.

The syntheses of the GO‐PVA/CS hydrogel (**Figure** **2**A) were based on previous reports^[^ ^29^ ^]^ with the addition of conductive filler. Briefly, GO and PVA dispersed in deionized water were heated and stirred to form the polymer precursor Solution A. Chitosan and metformin were dissolved in deionized water to form Solution B. Solutions A and B were then mixed at a 1:1 ratio at room temperature and poured into a mold to form the target hydrogel (Figure 2B and see Experimental Section for details).

We evaluated the electrically‐triggered release of metformin from GO‐PVA/CS hydrogel in phosphate buffered saline (PBS, pH 7.5). Customized test fixtures with titanium mesh electrodes connected to an external DC power were used to supply uniform voltage to the hydrogel (Figure S1, Supporting Information). The drug concentrations at different time points were measured through aliquots with an ultraviolet‐visible (UV‐vis) spectrophotometer at 233 nm (see Experimental Section for details). Compared to spontaneous release, the slope of the drug release curve with a 1 V voltage input was significantly larger, demonstrating that external DC voltage can accelerate drug release from GO‐PVA/CS hydrogel (Figure 2C).

We then switched the voltage on and off in intervals of 15 min for a total of 180 min, during which the drug release profile exhibited a controllable stepped pattern (Figure 2D). We speculated that this was due to an increase in the hydrogel's pore size as an external voltage was applied, which enhanced the free volume of the hydrogel matrix and thus accelerating drug release.^[^ ^21^ ^]^ An increase of DC voltage to 2 V led to faster release, peaking at 77% at 180 min compared to 54.1% for that of 1 V (Figure 2D). In addition, we conducted more detailed characterizations of the pharmacokinetic release of the hydrogel. The fitting results showed that the release model of the hydrogel was closer to the Higuchi model (Figure S2, Supporting Information). The Higuchi model, based on Fick's law, describes the diffusion process of water‐soluble/poorly soluble drugs in insoluble or soluble porous matrices. The Higuchi model suggests a diffusion‐controlled release mechanism, which is consistent with the properties of our hydrogel.

Next, we performed screening tests to compare the GO‐PVA/CS hydrogel with other biocompatible conductive hydrogels reported in the literature. Specifically, we chose carboxymethyl chitosan/citric acid (CMC/CA),^[^ ^30^ ^]^ poly(3,4‐ethylene dioxythiophene) (PEDOT),^[^ ^31^ ^]^ polyaniline/alginate (PANI/Alg),^[^ ^32^ ^]^ and polypyrrole‐polyacrylamide/chitosan (PPy‐PAM/CS),^[^ ^33^ ^]^ and tested their electrically‐triggered release of metformin (Figure S3, Supporting Information). Release ratio was defined as the average drug release rates when the voltage was on (triggered release), divided by the average drug release rates when the voltage was off (spontaneous release). Higher ratios indicated better selectivity with electrical triggers, while smaller error bars indicated better uniformity and durability. The GO‐PVA/CS hydrogel exhibited both a higher value and a smaller error bar during the 180‐min testing period (Figure 2E), and thus was chosen for subsequent studies.

We further tested the effect of pH on the drug release rate, and found that drug release was higher under neutral conditions (pH 7.5) compared to acidic and alkaline conditions (pH 5.5 and pH 9.5, Figure 2F). Figure 2F includes spontaneous release state (0 V) and accelerated release state (1 V). Analyzing each situation separately can provide a clearer explanation of the curve. We first separately displayed the accelerated release state (1 V) in Figure S4 (Supporting Information). An increase in pH promotes the ionization of carboxyl groups on graphene oxide, thereby adsorbing positively charged metformin and reducing the release of drug.^[^ ^34^, ^35^ ^]^ On the other hand, a decrease in pH reduces the ionization of graphene oxide, which in turn lowers the conductivity of the hydrogel and reduces the electro‐accelerated effect.^[^ ^36^ ^]^ Therefore, both acidic and alkaline conditions reduce the drug release rate. We then separately displayed the spontaneous release state (0 V) in Figure S5 (Supporting Information). Under acidic conditions, the amino groups (NH3⁺) in chitosan become protonated that lead to interactions between polymer chains and slight swelling of the hydrogel, thus accelerating the drug release.^[^ ^37^ ^]^ Under alkaline conditions, the deprotonation state of graphene oxide allowed for a gradual binding of ionized groups with the drug, leading to a gradual decrease in the drug release rate over time.^[^ ^35^, ^36^ ^]^ Therefore, the drug release curve under alkaline conditions was higher than that under acidic conditions in the short term, while in the long term, the trends of both conditions tended to converge. In summary, compared to spontaneous release state (0 V), the drug release rate during the accelerated release state (1 V) was more impacted by variations in pH.

Finally, we explored the influence of hydrogel compositions on drug release, and found that the release rate can be tuned by varying the CS and GO contents (Figure 2G,H; Figure S6, Supporting Information). This is mainly because chitosan can modify graphene oxide through non‐covalent methods, and the crosslinking between chitosan and graphene oxide enhances the hydrogel's stability, biocompatibility, and mechanical properties. This helps to increase the drug‐loading capacity of the hydrogel and improve the efficiency of electrically controlled drug release.^[^ ^38^ ^]^

**Figure 3:**
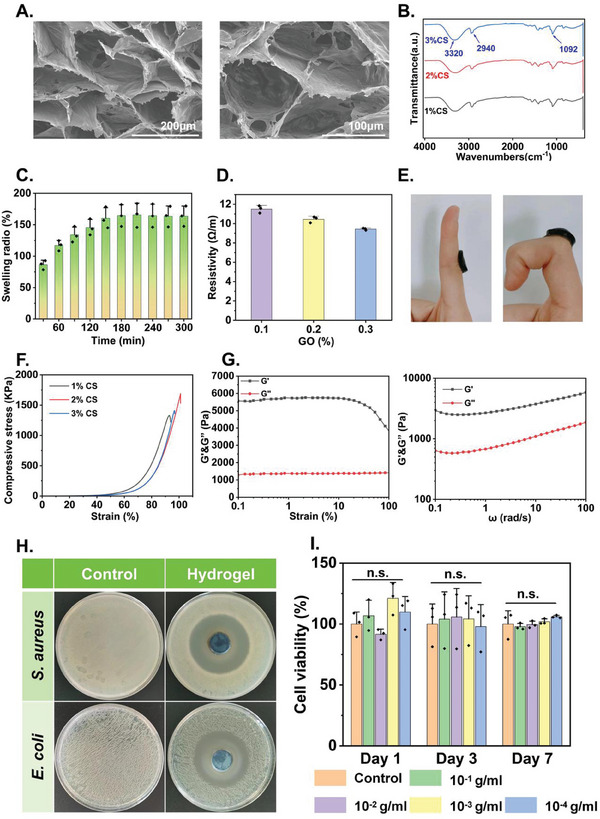
A) SEM images of the hydrogel at the scales of 200 and 100 µm. B) FTIR spectra of hydrogels with different CS concentrations. C) Swelling behavior of the hydrogel within 300 min, *n* = 3. D) Hydrogel conductivity with different GO concentrations. E) Images of morphological changes of the hydrogel applied on joints during movement. F) Strain curves of hydrogels with different CS concentrations. The peak indicates the onset of plastic deformation. G) Rheological curves of hydrogel's energy storage modulus (G') and loss modulus (G") varies with stress and frequency. H) Images of antibacterial experiment. The annular antibacterial zone formed around the hydrogel indicates its antibacterial properties. I) Biocompatibility of the hydrogel. Cell viability maintained ≈100% for 7 days under different hydrogel extracts concentration. And there was no significance between control group and other groups. Data are shown as mean ± SD, *n* = 3.

We performed a series of bench tests to characterize the GO‐PVA/CS hydrogel. Scanning electron microscope (SEM) images showed the porous structure of the hydrogel with an average pore size of 100 µm (**Figure** **3**A). Fourier‐transform infrared spectroscopy (FTIR) revealed distinct characteristic peaks of the hydrogel, particularly at 3320 cm−¹, indicating the ‐OH stretch of intermolecular and intramolecular hydrogen bonds (Figure 3B). The characteristic peak of the alkyl group observed at 2940 cm−¹ was attributed to the ‐CH stretch, while the main absorption peak at 1092 cm−¹ was attributed to the ‐C─O‐ stretch, confirming the formation of intermolecular hydrogen bonding between PVA, CS, and GO. Swelling test revealed that the hydrogel's weight increased by ≈100% within the first 180 min and remained stable afterward (Figure 3C), which facilitated the addition of drug solutions, and maintenance of a moist microenvironment at the wound site. Electrical resistivity of the hydrogel reduced as the GO content increased (Figure 3D). Experimental details of the above experiments can be found in the Experimental Section.

The bio‐adhesive ability of wound dressings is essential to prevent accidental peeling and promote healing during wound management. The hydrogel demonstrated good self‐adherence to human skin and could accommodate large movements such as 90° deformation (Figure 3E). The primary adhesion mechanism of the GO‐PVA/CS hydrogel to the skin relies on interfacial interactions between the hydrogel and skin tissue, including hydrogen bonding, host‐guest interactions, and molecular topological mechanisms. In addition, chitosan, the main component of the GO‐PVA/CS hydrogel, is a positively charged biopolymer. While generating topological entanglement with the skin, it can also enhance adhesion to the skin through electrostatic interactions. Mechanical tests revealed that the hydrogel can withstand more than 80% uniaxial compressive strain before plastic yield that is sufficient for most wound management scenarios (Figure 3F). The compression measurement model is shown in Figure S7 (Supporting Information). Furthermore, rheology tests revealed that the absolute values of the hydrogel's energy storage modulus (G′) and loss modulus (G″) were small, indicating that the material has high flexibility and low viscosity (Figure 3G). Curves of G′ and G″ did not intersect, indicating that the hydrogel did not flow under any strain conditions and therefore had strong morphological retention ability. From the frequency spectrogram (Figure 3G, **right panel**), it can be seen that G′ consistently exceeded G″ across the entire tested frequency range, which substantiated good elastic property of the material. Experimental details of the above experiments can be found in the Experimental Section.

We also characterized other mechanical properties of the hydrogel. In the uniaxial tensile experiment, we evaluated the tensile response of hydrogels with different chitosan concentrations by testing their stress‐strain curves. The samples were shaped like dumbbells (5 mm × 30 mm × 3 mm) and subjected to tensile tests at a constant speed of 5 mm min^−1^. The experimental results showed that the tensile strength of the hydrogels increased with the chitosan content, while the mechanical properties exhibited a trend of first increasing and then decreasing (Figure S8, Supporting Information). In the in situ lap‐shear test, to simulate real application scenarios, we placed the hydrogel between two pigskin samples for the experiment. The hydrogel was found to have a certain degree of self‐adhesive shear force (Figure S9, Supporting Information). In the peel test, we used a 90° peel test. The adhesive strength was determined by measuring the force required to peel the hydrogel from the adhesive interface. Pigskin was used as the substrate to simulate real application scenarios. A control group was set up with hydrogels coated with simulated tissue fluid on the surface to further verify the effect of tissue fluid on the adhesive performance of the hydrogel. The experimental results indicated that the presence of tissue fluid slightly reduced the adhesive performance of the hydrogel (Figure S10, Supporting Information).

The antimicrobial properties of hydrogels are also essential for preventing bacterial infections during wound management. We used *S. aureus* (gram‐positive bacteria) and *E. coli* (gram‐negative bacteria) to evaluate the antibacterial properties of hydrogels. As shown in Figure 3H, distinct antibacterial zones indicated good antibacterial properties to both *S. aureus* and *E. coli*. According to the area of antibacterial zones, the hydrogel exhibited a stronger antibacterial effect against *S. aureus* compared to that of *E. coli*. Experimental details of the above experiments can be found in the Experimental Section. We also verified the antibacterial properties of CS, the main component of the hydrogel. Figure S11 (Supporting Information) shows that there is a noticeable inhibition zone around the CS compared to the PBS control group, indicating that chitosan indeed has antibacterial properties.

Finally, we tested the biocompatibility of the hydrogel using a standard Cell Counting Kit‐8 (CCK‐8) analysis. As shown in Figure 3I (see Experimental Section), on day 1, 3, and 7, the cells maintained high viability at different concentrations of hydrogel extracts without significant difference to the control group, indicating no apparent toxicity of hydrogel. Experimental details of the above experiments can be found in the Experimental Section. We further evaluated the biocompatibility of the material by attaching it on the forearms of volunteers, with PDMS and commercial double‐sided adhesive as control, for up to 24 h to assess the degree of skin irritation. The results demonstrated lack of skin irritation of the hydrogel after long‐term wearing (Figure S12, Supporting Information).

After identifying the materials for drug loading, a pair of titanium mesh electrodes, customized wireless flexible circuits, and a soft chassis consisting of PDMS were designed and assembled to form the final hydrogel bioelectronics. The entire device measured 4.5 cm by 2 cm by 0.5 cm and can hold up to 2 mL of drug solution.

**Figure 4:**
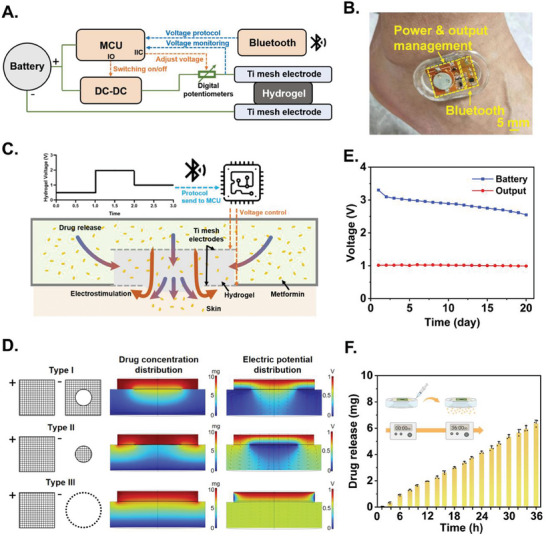
A)Schematic diagram of the chip circuit. Green lines demonstrate the connection of power. Orange lines demonstrate the MCU control signal. Blue line shows the input information to MCU. B) Image of the device adhered to ankle, featuring the MCU module (with Bluetooth and antenna), the voltage control module (DC–DC for voltage regulator and digital potentiometer for voltage adjustment) and the battery. Scale bar: 5 mm. C) Working principle of the device. After receiving the protocol, the MCU controlled the voltage on titanium mesh electrodes. The blue–red gradient arrow indicates the direction of accelerated drug diffusion. The red arrow indicates the direction of the voltage drop caused by electrostimulation. D) Simulation of different types of titanium net electrodes. Type I was a complete piece closely attached to the bottom of the hydrogel, with a circular window in the middle exposing the hydrogel to contact the wound. Type II was a single small round piece attached to the bottom center of the hydrogel. Type III wrapped around the side of the hydrogel, allowing the hydrogel to completely contact the wound. The drug concentration distribution and potential distribution are both steady‐state results. In the drug concentration distribution, red represents high concentration, blue represents low concentration, and gradient colors represent changes in concentration gradients. The larger the gradient area, the more uniform the drug release. In the potential distribution, red represents high potential, blue represents low potential, and gradient colors represent changes in potential gradients. The red arrow indicates the direction of the potential at that point. E) Voltage change of battery and titanium mesh electrodes over days; F) Refilling test result of the device. Data are shown as mean ± SD, *n* = 3.

The circuit consisted of a MCU that incorporated Bluetooth for wireless communication (**Figure** **4**A). Photo of the completed circuit and device worn on the ankle where diabetic wounds frequently occur is shown in Figure 4B. The preset voltage protocol can be sent to the circuit via Bluetooth from a mobile phone app or a computer. The digital potentiometer functioned as a current‐limiting resistor, and its resistance value can be adjusted via the Inter‐Integrated Circuit (IIC) interface to control the voltage applied to the titanium mesh electrodes. The power supply can also be turned off by controlling the DC–DC regulator. The MCU continuously monitored the voltage of the titanium mesh electrodes through analog‐to‐digital acquisition and adjusted the digital potentiometer to maintain stable voltage supply. The circuit was powered by a 3 V lithium button battery.

Figure 4C demonstrates the working principle of the device. The wireless circuits were designed such that it not only allowed preset voltage protocols, but also supported real‐time voltage adjustments as needed. As the voltages were turned on and off on‐demand and wirelessly via the MCU, release kinetics of metformin can be accelerated or decelerated in accordance with the pathological rhythms. Meanwhile, the titanium mesh electrodes provided programmable electrostimulation at the wound site through the hydrogel, which also promoted wound healing.

The configuration of the titanium mesh needs to ensure both sufficient contact between hydrogel and wound and an adequate electric field gradient within the hydrogel to maximize the effect of drug release acceleration. To optimize the titanium mesh configuration, we used COMSOL simulation software package to simulate the drug release gradient and the electric field distribution under three different types of titanium mesh configurations (Figure 4D). Although Type III had the best distribution uniformity in terms of drug release, the electric field gradient inside the hydrogel was concentrated on the side, which failed to effectively accelerate drug release and provide electrostimulation at the center of the wound. In Type II, the electric field gradient distribution inside the hydrogel was widespread, providing a better acceleration effect on drug release, but the drug release gradient cannot cover the majority of wound area. Comparatively, Type I not only can provide smooth drug release during electrostimulation, but also can form electric field gradient that was centered around the wound. We thus chose Type I electrodes during actual device implementation.

To verify the long‐lasting characteristics of the device, we tested the circuit's endurance and the refillability of the drug. We characterized the circuit's endurance by recording battery output voltage and the voltage output by the titanium mesh electrodes. After 20 days of continuous operation, the battery output voltage can still support circuit functions and voltage on electrodes remained stable at 1 V, indicating that the device can endure at least 20 days without battery changes (Figure 4E). For refillability, we conducted bench tests using a simplified, 3D printed testing fixture that simulated real device implementation on real skin (Figure S13, Supporting Information). The results showed that with a single refilling of 2 mL of 3% metformin, the new drug solution quickly permeated and replenished the hydrogel during the initial 3–4 h and then began to release for at least 36 h (Figure 4F). We further tested sealing property of the device, where the drug‐containing device was completely immersed in water, and no significant increase in drug release was found, indicating that the device has good sealing properties against shower, rain, or other scenarios during actual use (Figure S14, Supporting Information). At the same time, we verified the sealing of the device under mechanical deformations, and the results demonstrated the viscoelasticity of the PDMS was sufficient to avoid leakage from the pinholes after syringe injection (Movie S1, Supporting Information).

Building upon the aforementioned work, we further explored the performance of the device. First, we simulated human motion to investigate whether the device can function normally during typical human movement. The results showed that when the device was bent or twisted, there was a small voltage fluctuation that did not affect the electrostimulative performances (Figure S15 and Movie S2, Supporting Information). The drug release under large deformation slightly increased compared to the stationary state in the early stages of the experiment, while the drug release trends gradually aligned as the experiment progressed (Figure S16, Supporting Information). We also explored whether it was possible for the device to combine alternating current (AC) and DC stimulation to expand the modality of electrostimulation. We set the AC voltage at 10 Hz, 50% duty cycle, cycling once every 30 min through 1 Vpp, 2 Vpp, and 0 Vpp. The curves showed that the release slope of the AC voltage is slightly lower than that of the DC voltage, but higher than that of spontaneous release (Figure S17, Supporting Information). This indicates that, like DC voltage, AC voltage also accelerates the drug release from the hydrogel, but the effect is slightly lower due to the presence of duty cycles, and the contrasting between ON and OFF states are less significant. Finally, we explored battery‐less, wireless operation of the device. Inspired by many recent reports on bioelectronics powered by radio‐frequency (RF) power transmissions, we removed the battery and replaced it with a receiving coil and rectifier circuit. To minimize the size, the rectifier circuit adopted a half‐bridge design, consisting of rectifier diodes, voltage‐limiting diodes, filtering capacitors, and voltage‐shaping capacitors. We measured the wireless power transmission distance, the rectified voltage at the coil receiver, and the voltage across the electrodes of the hydrogel. Wireless charging can be achieved within a distance of 3–7 mm (Figure S18, Supporting Information). For future optimization, the antenna frequency can be fine‐tuned to 13.56 MHz using antenna design to enable power transmission over longer distances.^[^ ^39^, ^40^ ^]^

**Figure 5:**
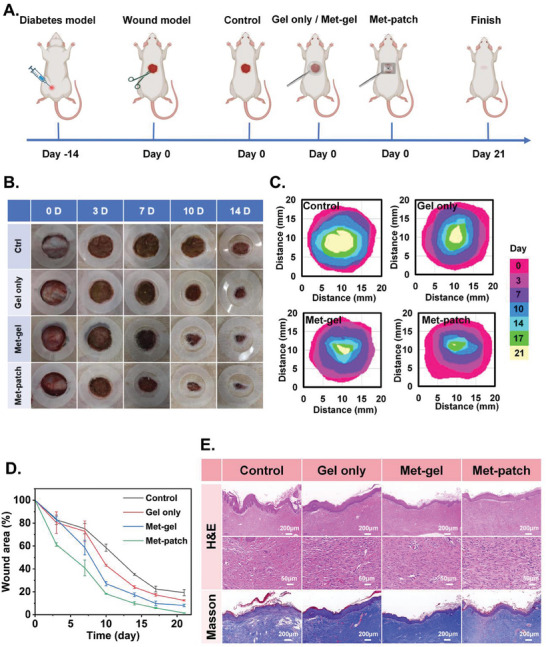
A) Schematic diagram of the experimental procedure. B) Representative images of the wound area collected from the four groups on days 0, 3, 7, 10, and 14. C) Wound residual area measured by ImageJ software on days 0, 3, 7, 10, 14, 17, and 21. D) Rate of wound closure in rats over 21 days. E) H&E staining and Masson staining at the wound site on day 21. Scale bars are 200 and 50 µm. Data are shown as mean ± SD, *n* = 3.

Finally, we evaluated the efficacy of the device for diabetic wound healing in diabetic rat models. We induced diabetes in rats 14 days prior to creating the wounds and stabilized their blood glucose levels above 16.7 mmol L^−1^, which were accompanied by symptoms of polydipsia, polyphagia, and polyuria. A circular wound with a diameter of 1.8 cm was then created on the back of the rats (see Experimental Section for details). The samples were secured to the rat using a motion bandage and medical adhesive tape. To prevent the rats from biting the surface, a bitter nail solution was applied. The hydrogel dressing was replaced every five days to facilitate wound aeration, and the drug solution in the device was refilled at the same interval. The rats were divided into four groups: self‐healing (control), blank hydrogel (gel only), hydrogel with metformin (met‐gel), and hydrogel bioelectronics with metformin (met‐patch) (**Figure** **5**A).

Photos of the wounds were collected on days 0, 3, 7, 10, 14, and 21 and analyzed for reductions in wound area (Figure 5B; Figure S19, Supporting Information). It can be found that the control group showed the slowest healing of wounds and barely heal after 21 days. The gel only group demonstrated a slightly better therapeutic effect than the control group, verifying that the antibacterial property of hydrogel alone has contributed to wound healing. The met‐gel group showed a significant improvement over the blank hydrogel, confirming the efficacy of metformin in treating diabetic wounds. Finally, the met‐patch group exhibited the most impressive treatment outcome characterized by the fastest descending gradient of average wound diameters, as well as the smallest remaining wound area at each day. No signs of redness, swelling, or infection were observed (Figure 5C,D). After a 21‐day treatment, the wound healing rates were 1.5% ± 0.25%, 8.1% ± 1.11%, 12.5% ± 0.6%, and 19.3% ± 2.6% for the four groups, respectively (Figure 5D).

Histological and immunochemical analyses were further performed on day 21 after the animals were sacrificed to assess wound healing progression and skin quality. H&E staining revealed different degrees of granulation tissue growth among different experimental groups. Specifically, the met‐patch group exhibited the shortest wound length and a complete epithelialization (Figure 5E, **top row**). Zoom‐in evaluation revealed more tightly packed fibroblasts, increased neovascularization, and new epidermis in the experimental group (Figure 5E, **middle row**). Masson staining was used to observe collagen distribution across different treatment groups as a clinical standard for assessing wound healing. In the met‐patch group, collagen fibers were neatly arranged and showed a well‐structured hair follicle formation (Figure 5E, **bottom row**). These results have indicated that a synergetic effect from electrostimulation, drug, and hydrogel dressing can accelerate diabetic wound healing. We conducted additional immunofluorescence staining experiments on diabetic wounds of the rats on day 21 to further evaluate angiogenesis and inflammatory response at the wound site. We selected interleukin‐6 (IL‐6) to characterize the inflammatory response of the wound and cluster of differentiation 31 (CD31) Cell Counting Kit‐8 to represent neovascularization.^[^ ^41^, ^42^ ^]^ The results show that the met‐patch group has a higher level of neovascularization compared to the control group, while the inflammatory markers are lower than those in the control group. These have indicated that the hydrogel patch led to reduced inflammation and enhanced neovascularization, thereby improving the healing process of diabetic wounds (Figure S20, Supporting Information).

## 3 Conclusion

In conclusion, we have developed a set of fabrication and integration schemes for wireless, programmable, and refillable hydrogel bioelectronics that show accelerated wound healing in diabetic rat models. GO‐PVA/CS hydrogel was chosen due its mechanical robustness, biocompatibility, and anti‐bacterial property, and its electrically triggerable drug release performance was benchmarked against other conductive hydrogels in the literature. A flexible and wireless circuit incorporating low power consumption, wireless communication, and voltage regulation was designed and fabricated to allow watertight integration with the hydrogel, leading to programmable drug release and electrostimulation at the wound sites. In vitro and in vivo studies confirmed that the device provided both sustained and programmable metformin delivery and electrostimulation for up to 21 days in diabetic rat models and accelerated wound healing compared to control groups.

The results of this study substantiate a promising direction that is to combine conventional pharmaceuticals with programmable electrostimulation for advanced wound management and potentially other conditions. A possible future direction is to integrate digital physiological sensors, such as blood glucose monitoring in this case, with real‐time data readout to form a closed‐loop, responsive voltage control algorithm with the hydrogel bioelectronics to further enhance diabetic‐related therapeutics.

## 4 Experimental Section

### Materials

Chitosan (Deacetylation degree 95%, Macklin), PVA (Mw ≈205000, Macklin), graphene oxide (Macklin), acetic acid (Macklin), metformin hydrochloride (98%, Macklin), PBS (pH 7.5, Biosharp), Cell Counting Kit‐8 (CCK‐8) (Yeasen), Roswell Park Memorial Institute (RPMI) 1640 (Gibco), Fetal Bovine Serum (Sigma), Penicillin‐Streptomycin Liquid (Solarbio), Streptozocin (STZ) (Solarbio) were purchased directly from the above vendors and used as received.

### Syntheses of the GO‐PVA/CS Hydrogel

Hydrogel preparation was performed according to the previously reported method.^[^ ^29^ ^]^ A concentration of 0.2% GO was dispersed in deionized water, with 9% polyvinyl alcohol added, then heated and stirred at 95 °C for 5 h to form polymer precursor solution A. Chitosan powder was dissolved in 1% acetic acid to prepare a chitosan solution at a 2% concentration, forming solution B. At room temperature, solutions A and B were mixed in a 1:1 ratio to form a gel solution. The solution was poured into a mold, and frozen at −20 °C for 4 h, and this process was repeated three times to obtain the target hydrogel. For the drug‐loaded hydrogels, the model drug metformin was added to the preformed hydrogel solution by active loading at a concentration of 3 mg mL^−1^.

### Device Fabrication

The 3D modeling of the device and the mold structure was designed using SolidWorks. The stainless‐steel mold was processed using Computer Numerical Control (CNC) machining. PDMS (SYLGARD 184, Dow Corning) with an 8% curing agent ratio was degassed for 20 min and then poured into the mold. The mold was placed on a hot plate at 110 °C to cure for 30 min before demolding. After treating the PDMS surface with primer (LOCTITE SF 7701), the titanium mesh was bonded to the PDMS using instant adhesive (LOCTITE 4011), and the hydrogel was encapsulated within the device. The flexible circuit was designed using PADS and manufactured with FPC process. The titanium mesh electrodes and flexible circuit were connected using enameled wire through welding. The microcontroller and current‐limiting resistor were soldered to the enameled wire. Simulations were performed using COMSOL Multiphysics. The concentration distribution simulation used the Transport of Diluted Species interface, and the electric field distribution simulation used the Electric Currents interface. Both results were solved in steady‐state.

### Drug Release Curve Measurement

The hydrogel with an initial loading concentration of 3 mg mL^−1^ was immersed in 50 mL of phosphate buffer solution (PBS, pH 7.4), and voltage was applied to the hydrogel using a double electrode system at 37 °C. An aliquot of the solution was removed at predetermined intervals and replaced with an equal amount of PBS to maintain a constant volume. The drug concentration was measured at 233 nm using a UV–vis spectrophotometer. Three parallel experiments were conducted, and the cumulative drug release was calculated using the formula: DL = (A/A_0_) × 100, where A is the drug content of the sample measured by the UV–vis spectrophotometer, and A_0_ is the initial drug load of the hydrogel. The voltage output of the device was pre‐set according to the testing mode, either through a program or wireless commands. Drug release was tested using the same protocol as above. In experiments requiring repeated drug supplementation, an additional syringe was used to replenish the drug solution into the device.

### Hydrogel Morphology

Morphology of the hydrogel was observed using a desktop SEM (SU‐3500). The freeze‐dried hydrogel was sectioned, and its lyophilized structure was examined after sputter coating.

### Fourier Transform Infrared (FTIR) Spectrum

Structural analysis of the hydrogel was performed using FTIR spectroscopy. Hydrogels containing 1%, 2%, and 3% (w/v) chitosan were freeze‐dried, and their components were scanned in the wavelength range of 4000 to 500 cm−¹ using FTIR spectroscopy (Nicolet iS50, USA).

### Swelling Performance

Swelling of the hydrogel was determined using the gravimetric method. At 37 °C, the hydrogel was immersed in PBS, removed at specific time intervals, blotted with filter paper to remove surface moisture, and weighed with an analytical balance to determine the swelling rate. The equilibrium swelling ratio was determined when the hydrogel weight remained constant. Three parallel experiments were set up, and the results were averaged. The swelling ratio was calculated using the following formula: Swelling Ratio = (W_2 –_ W_1_)/W_1_ × 100%, where W_1_ and W_2_ are the weights of the initial and swollen hydrogels, respectively.

### Conductivity

Conductivity of the hydrogel was tested using a pair of titanium mesh electrodes closely attached to the upper and lower surfaces of the hydrogel, which were then connected to an impedance analyzer (TH2839, Tonghui) for impedance testing.

### Stress‐Strain Performance

Hydrogels were characterized for their mechanical properties using a universal material tester (Zwick/Roell Z020). In the compression test, the weighing sensor was set to 200 N, and the crosshead's working speed was set to 5 mm min^−1^. The compressive strength of the hydrogel was designated as the stress value when the hydrogel failed.

### Rheological Properties

A rotary rheometer (HAAKE MARS 60) was used to measure the rheological properties of the hydrogel and determine its viscoelasticity and mechanical properties. Strain scanning and frequency scanning were performed at 37 °C. The fixed frequency for strain scanning was 1 Hz with a stress variable range of 0.1–100%, while the frequency scan was set at 1% strain with a scanning frequency range of 0.1‐100 rad s^−1^.

### Antibacterial Properties

Gram‐positive bacteria (*S. aureus*) and Gram‐negative bacteria (*E. coli*) were used as experimental strains to evaluate the antibacterial efficacy of the hydrogels. Individual colonies of both bacteria were grown overnight at 37 °C in beef extract‐protein broth to yield a bacterial suspension. Before testing, all materials were sterilized using an autoclave. The bacterial suspension was diluted, and both bacteria were spread evenly on Luria Bertani (LB) agar at concentrations of 1 × 10⁸ CFU mL^−1^ and 1 × 10⁶ CFU mL^−1^. The sterilized hydrogel was then placed on the LB agar plates and incubated for 24 h at 37 °C to observe colony growth.

### Cytotoxicity Assay

*T*he cytotoxicity assay for the hydrogels was performed according to the protocol of the CCK‐8 kit. Sample extracts were prepared at a concentration of 1 g/10 mL and incubated for 24 h at 37 ± 1 °C in culture media (RPMI 1640; 10% Fetal Bovine Serum; 1% Penicillin‐Streptomycin Liquid). The extracts were then diluted with culture medium to various concentrations (100, 10, 1, and 0.1 mg mL^−1^). L929 cells were seeded in 96‐well plates at a density of 1000 cells per well (100 µL). After 24 h of culture, the medium was decanted, and 100 µL of the extracts were added to the 96‐well plates and incubated for 1, 3, and 7 days. The CCK‐8 solution was then added to each well and incubated for an additional 0.5–1 h. Absorbance was measured at 450 nm using a microplate reader (SpectraMax iD5, Molecular Devices).

### In Vivo Animal Experiments

Male Sprague‐Dawley (SD) rats, 6 weeks old (200–250 g), were used for the study. The rats were given a single intraperitoneal injection of 65 mg kg^−1^ streptozotocin (STZ) and monitored for 16 h. Three days later, blood glucose and weight were measured twice a week for a total of two weeks. Successful diabetic modeling was confirmed if blood glucose levels were consistently >16.7 mmol L^−1^ and accompanied by symptoms of polyphagia, polydipsia, polyuria, and weight loss. These diabetic rats were then used to establish the wound model.

Rats were anesthetized via intraperitoneal injection of pentobarbital (35 mg kg^−1^). Following anesthesia, diabetic rats were shaved, and a circular wound was created on the dorsal center of each rat. Subsequently, the rats were allocated into three experimental groups: blank hydrogel, liquid hydrogel dressing, and electro‐controlled hydrogel device, along with an additional blank control group. The hydrogel and device were fixed using medical tape and elastic bandages, with the hydrogel dressing being replaced every 5 days, while supplementing the liquid formulation into the device. During replacement, the medical tape and elastic bandages were removed, and the hydrogel dressing was gently peeled off and replaced with a new hydrogel, after which the medical tape and elastic bandages were re‐attached. The drug chamber filled with liquid constantly maintained the hydrogel in a moist state with relatively low adhesiveness, and the peeling force of the hydrogel was insufficient to cause secondary damage to the wound. Wound images and measurements were taken on days 0, 3, 7, 10, 14, 17, and 21, and the wound area was quantified using Image J. On day 21, the regenerated wound tissue was collected and fixed with paraformaldehyde for H&E and Masson staining to assess tissue growth.

After the experiment, the animals were humanely euthanized. The rats were first deeply anesthetized with an intraperitoneal injection of 50 mg kg^−1^ pentobarbital, followed by an air needle injection.

### Statistical Analysis

The data calculated in the experiment were all presented as mean ± SD, and each data quantitative evaluation was repeated at least three times. Two‐way ANOVA was performed in data on a quantitative dependent variable multiple levels of two categorical independent variables. All data were analyzed by Origin 2024 and Graphpad Prism 9.0.

### Ethics Statement

All animal experimental procedures were approved by the Animal Experimental Ethics Committee of Zhejiang University, and were performed in accordance with their guidelines and protocols (Approval Number: ZJU20240534).

The wearable devices developed in this study were tested on human volunteers (graduate students in the group) for up to 24 h for assessing wearability and bio‐compatibility. All participants were well‐informed of the details of procedures and potential risks by going through written documentations, and agreed prior to the tests. Since the experiments were non‐invasive with minimal risks, the institution (Zhejiang University) does not require formal ethic approval regarding these type of experiments.

## Conflict of Interest

The authors declare no conflict of interest.

## Supporting information



Supporting Information

Supplementary Movie 1

Supplementary Movie 2

## Data Availability

The data that support the findings of this study are available from the corresponding author upon reasonable request.
