# Supplementary material for: Wireless, Programmable, and Refillable Hydrogel Bioelectronics for Enhanced Diabetic Wound Healing
Source: Adv Sci (Weinh). 2024 Oct 14;11(45):2407820. doi: 10.1002/advs.202407820 (PMC11615824; doi:10.1002/advs.202407820)
Supplement: Supplementary file 1 — Supporting Information [file ADVS-11-2407820-s002.pdf]

ADVANCED SCIENCE
Open Access

Supporting Information

for *Adv. Sci.*, DOI 10.1002/advs.202407820

Wireless, Programmable, and Refillable Hydrogel Bioelectronics for Enhanced Diabetic Wound Healing

Ningjie Du, Yunlong Fan, Yunting Zhang, Hao Huang, Yidan Lyu, Ruisi Cai, Yuqi Zhang, Tianyuan Zhang, Yixin Guan and Kewang Nan**

Supporting Information for

Wireless, Programmable, and Refillable Hydrogel Bioelectronics for Enhanced Diabetic Wound Healing

Ningjie Du#, Yunlong Fan#, Yunting Zhang, Hao Huang, Yidan Lyu, Ruisi Cai, Yuqi Zhang, Tianyuan Zhang, Yixin Guan*, Kewang Nan*

#These authors contributed equally to this work.

*Corresponding authors

[*] E-mail: Kewang Nan – knan@zju.edu.cn

Yixin Guan – guanyx@zju.edu.cn

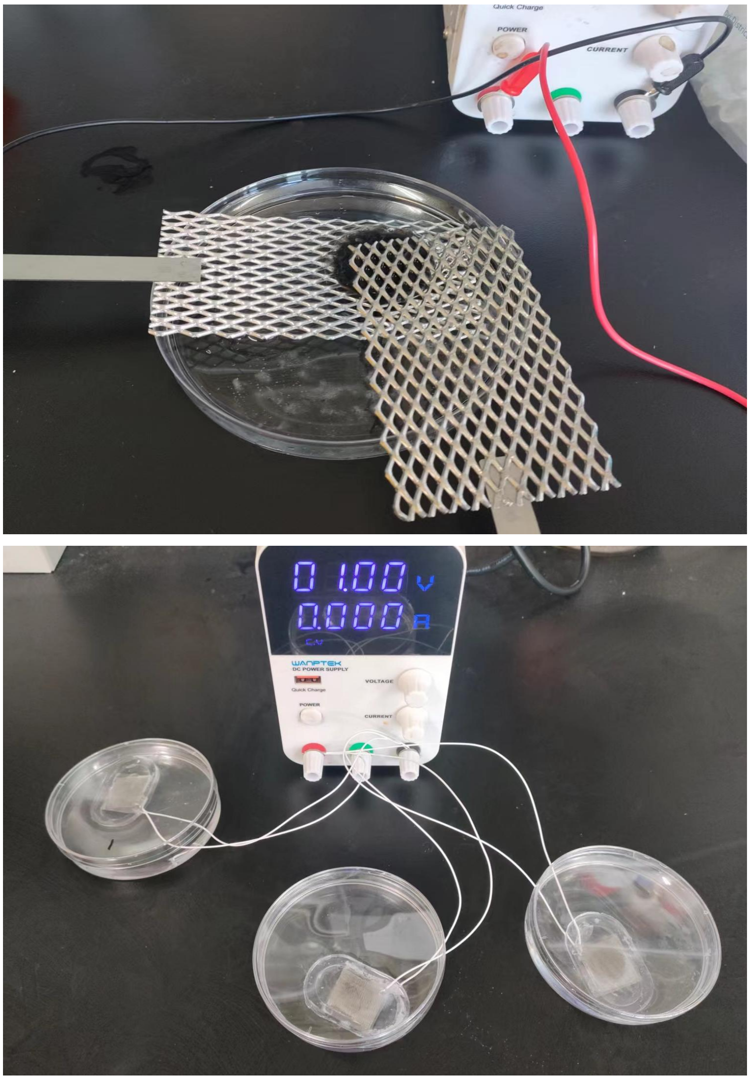

Figure S1. Dual-electrode system used for the drug release experiments.

Voltage	Kinetic models					
	Zero-order		First-order		Higuchi	
	K_0	R^2	K_1	R^2	K_H	R^2
0V	0.0097	0.9891	0.0030	0.9898	0.0973	0.9899
1V	0.0183	0.9460	0.0339	0.9941	0.1876	0.9957
2V	0.0244	0.9528	0.0386	0.9877	0.2498	0.9970

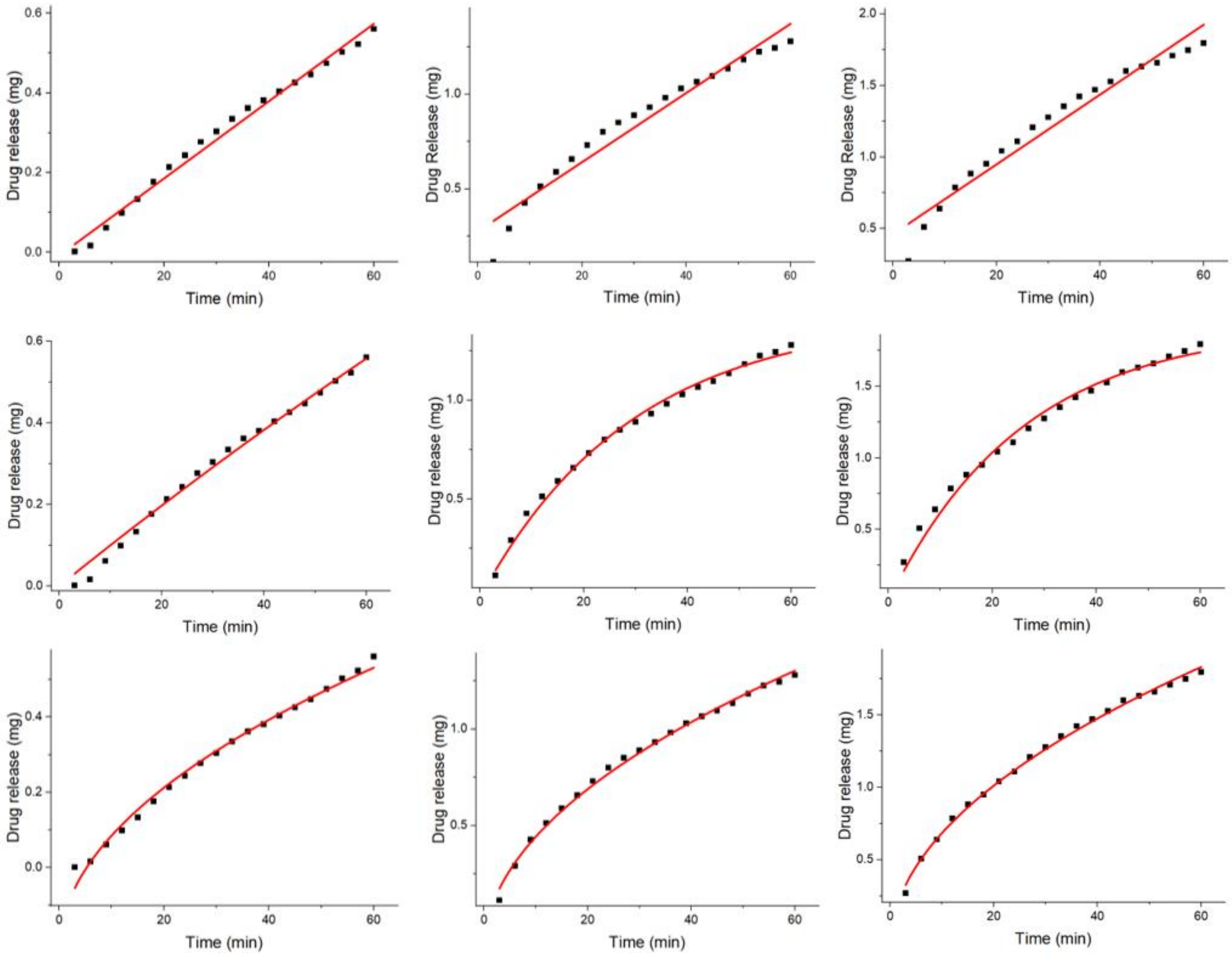

Figure S2. Pharmacokinetic release of the hydrogel.

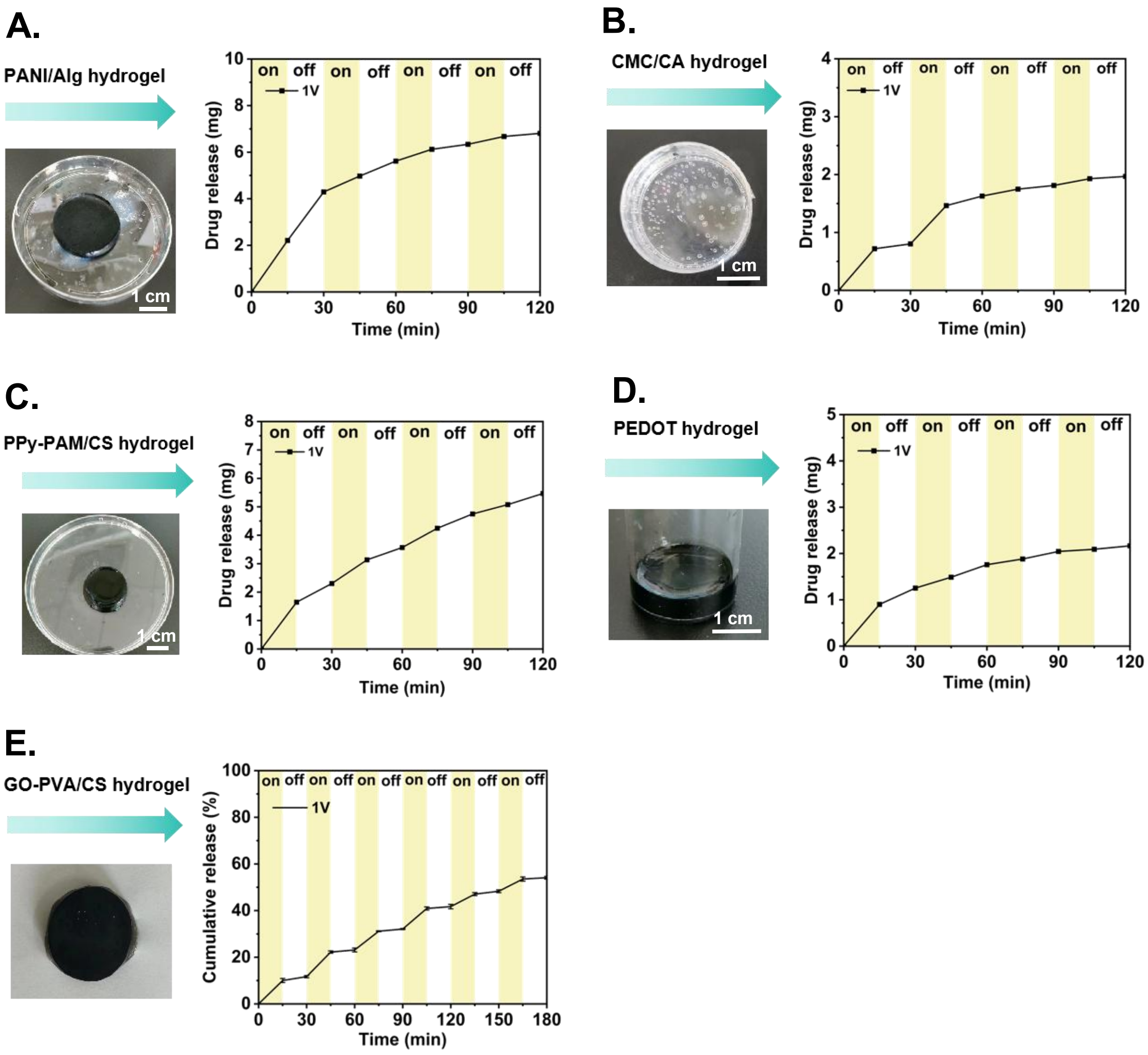

Figure S3. (A-E) Illustrates the release effect of different types of hydrogels on the loaded drug at 1V voltage, repeated every 15 minutes.

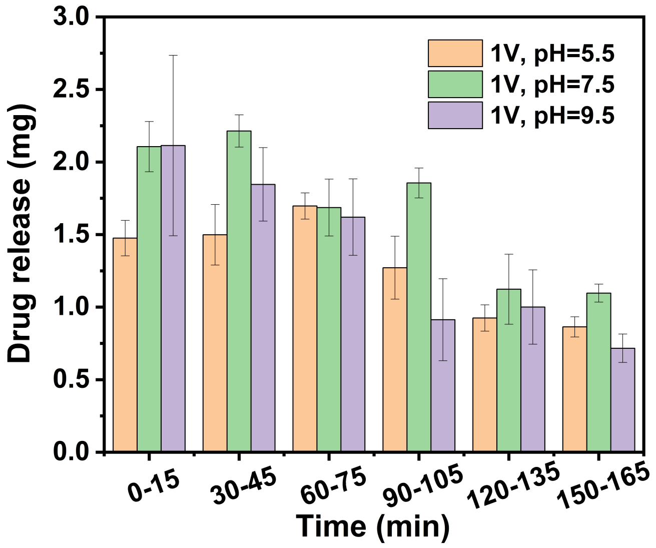

Figure S4. Drug release under different pH conditions during the voltage-on phase.

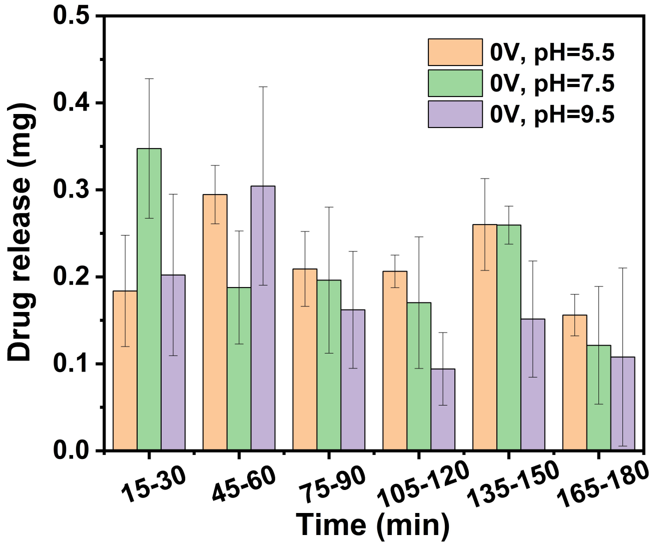

Figure S5. Drug release under different pH conditions during the voltage-off phase.

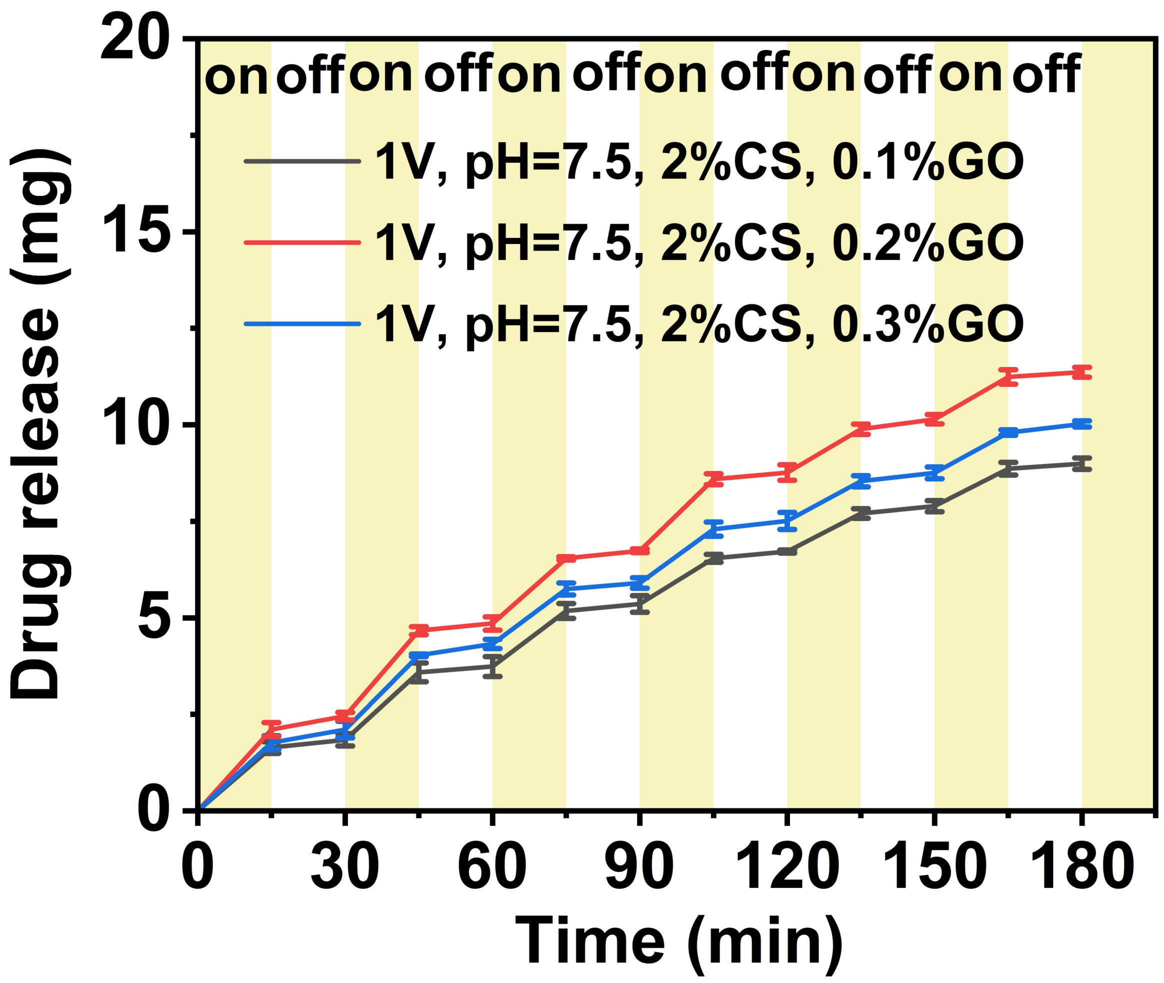

Figure S6. Drug release curves with different GO content with 15 min interval.

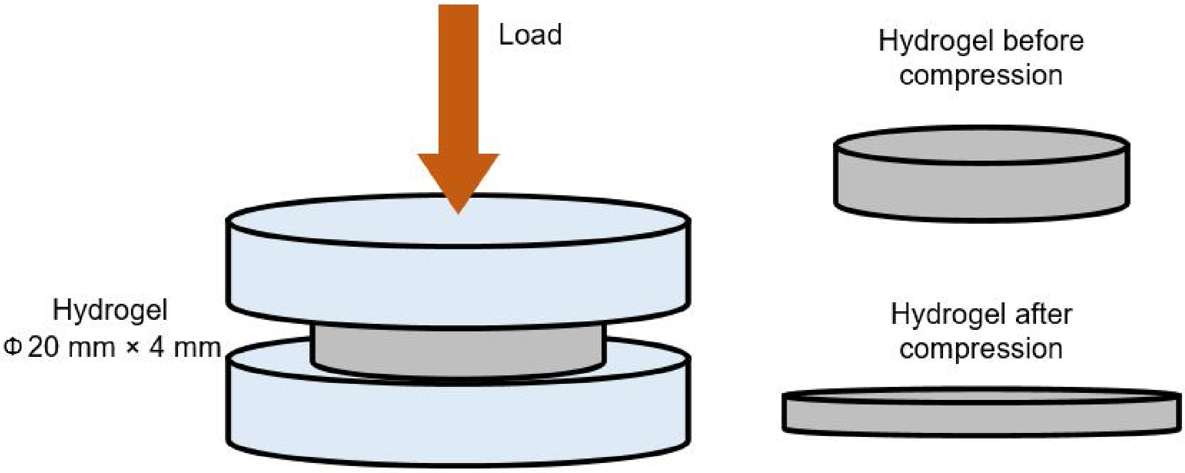

Figure S7. Compression measurement model of the hydrogel.

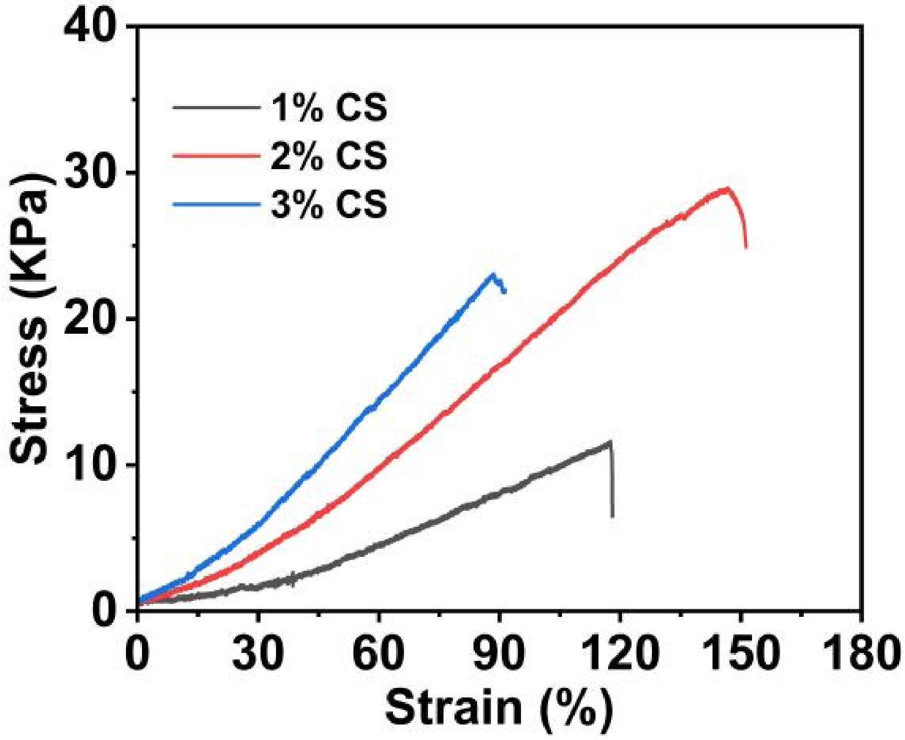

Figure S8. Elastic response of hydrogels at different CS concentrations.

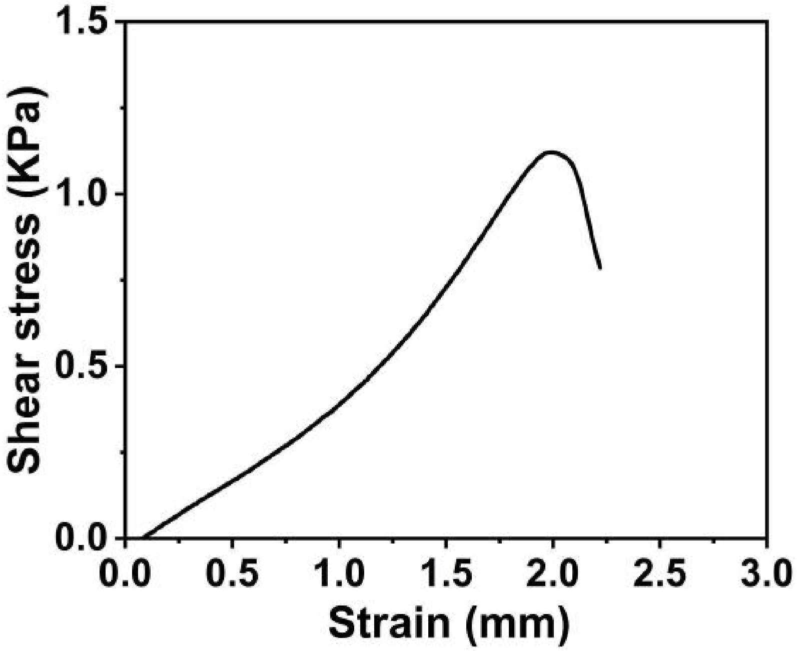

Figure S9. Self adhesive shear force of the hydrogel.

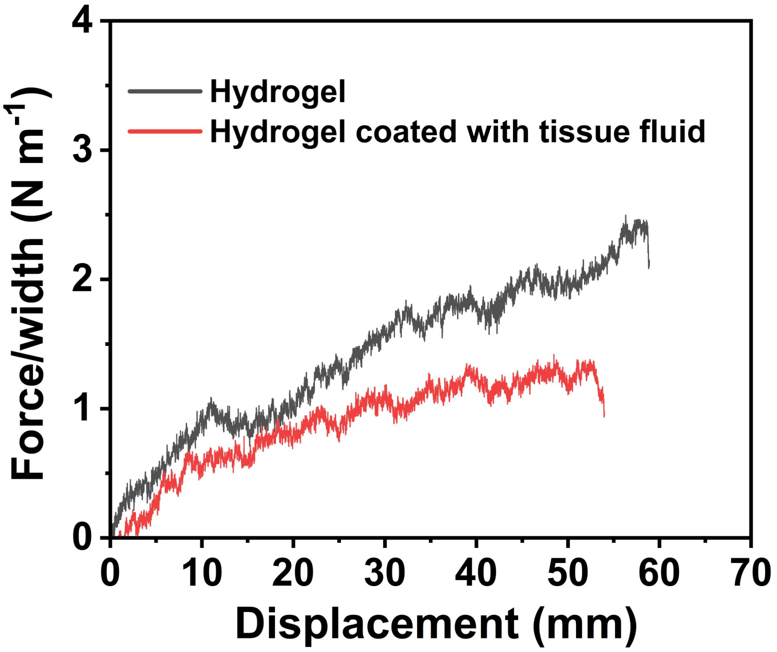

Figure S10. Peeling strength of hydrogels.

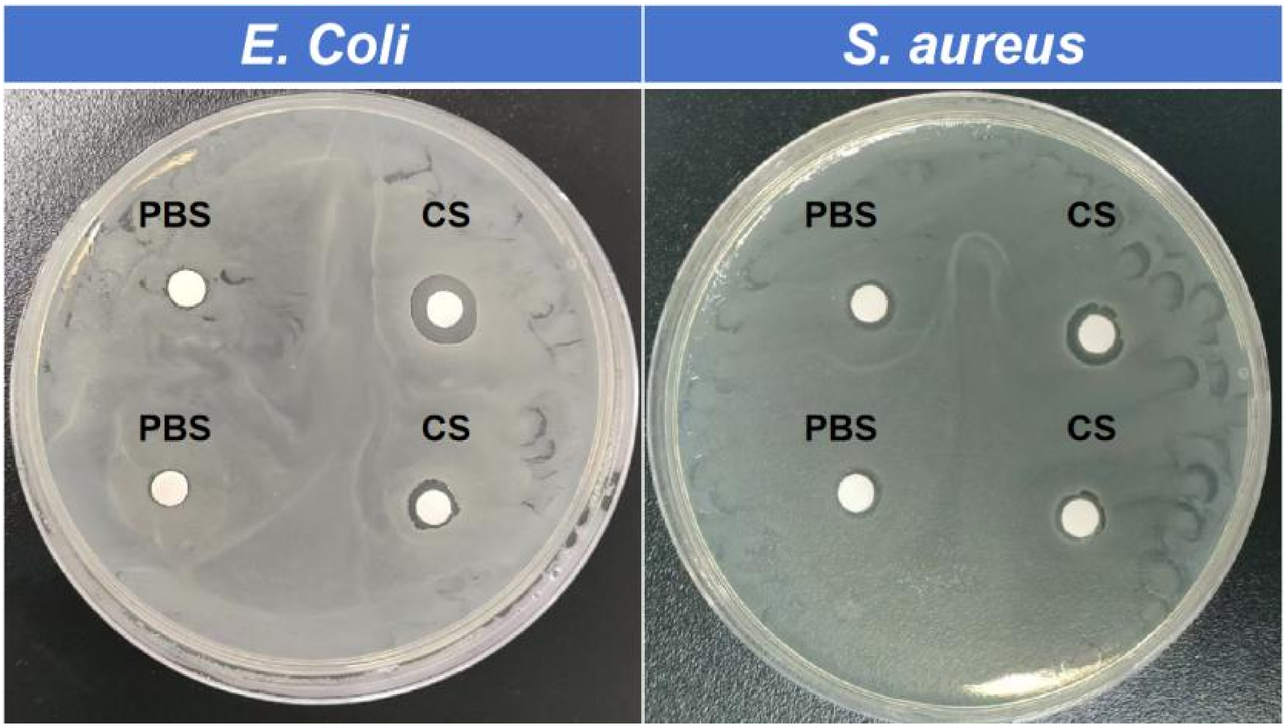

Figure S11. Antibacterial properties of chitosan.

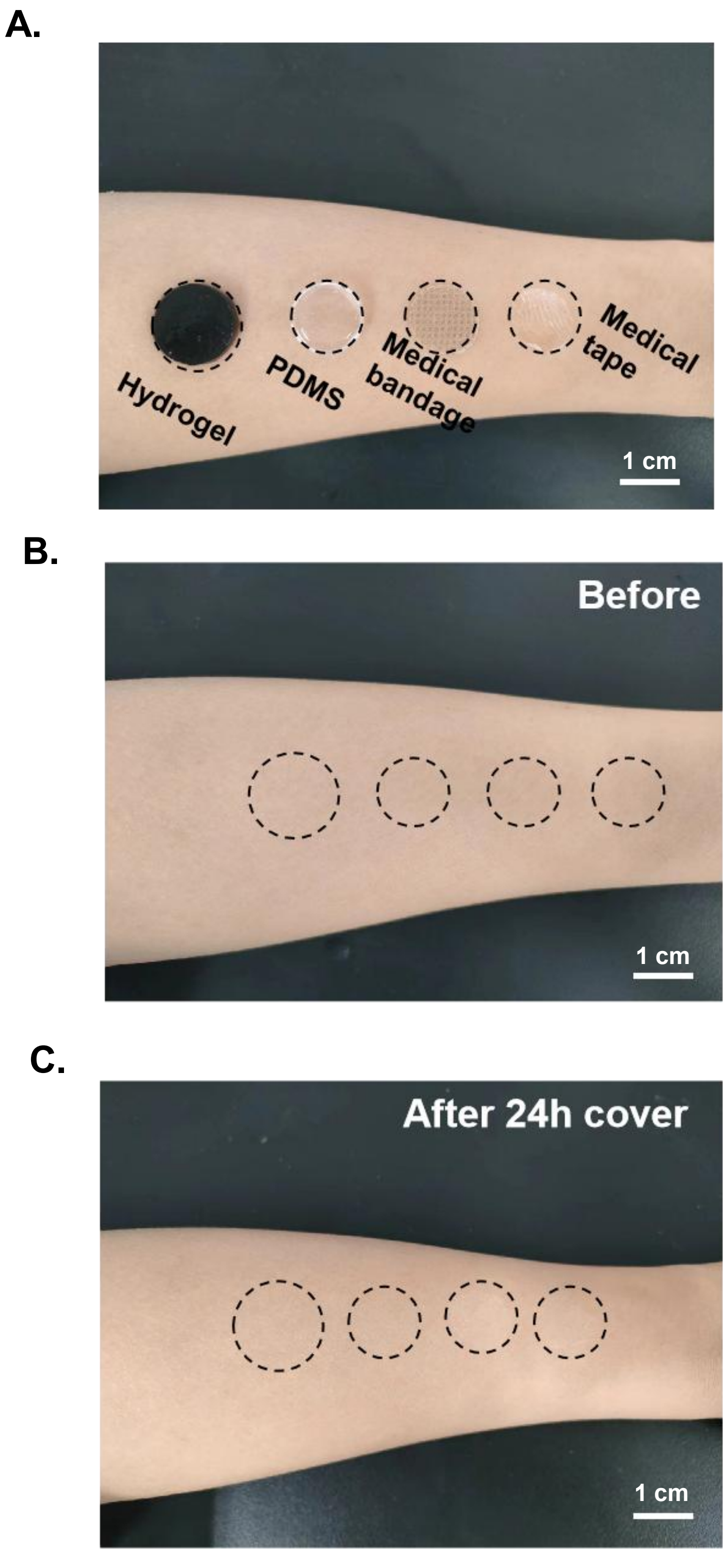

Figure S12. (A-C) Presents optical images taken before and after being covered for 24 hours with hydrogel, PDMS, medical bandage, and medical tape.

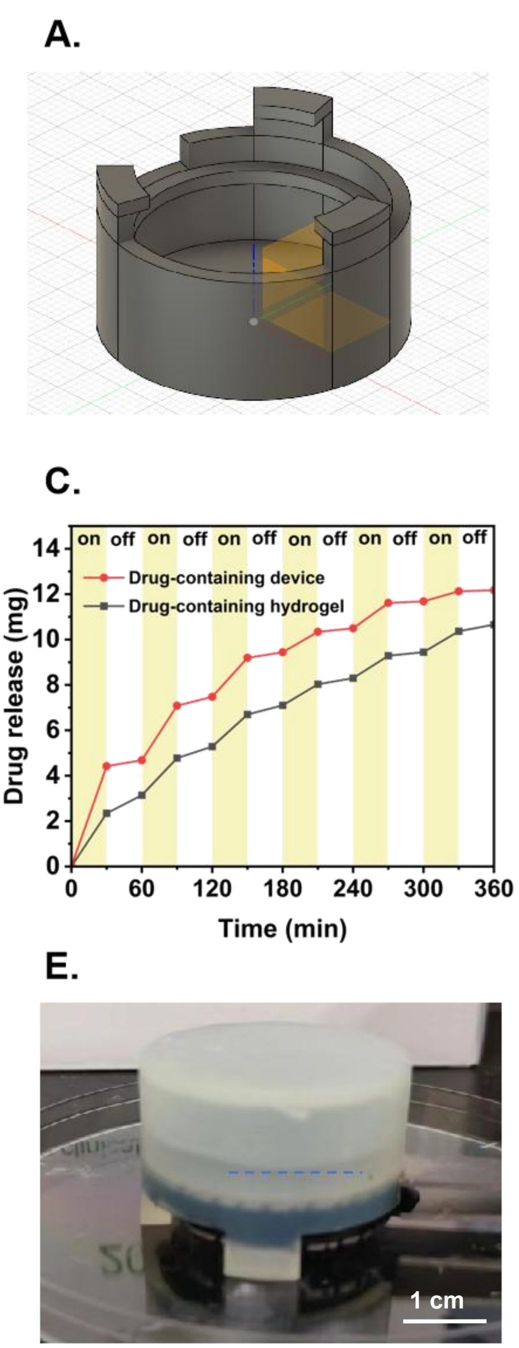

Figure S13. (A) The drug supplement device model. (B) The 3D printing device. (C) Drug release curves of drug-free device hydrogel and drug-containing device hydrogel at 1V, repeated every 30 minutes. (D-F) Status of the drug solution in the device at 0, 24, and 48 hours.

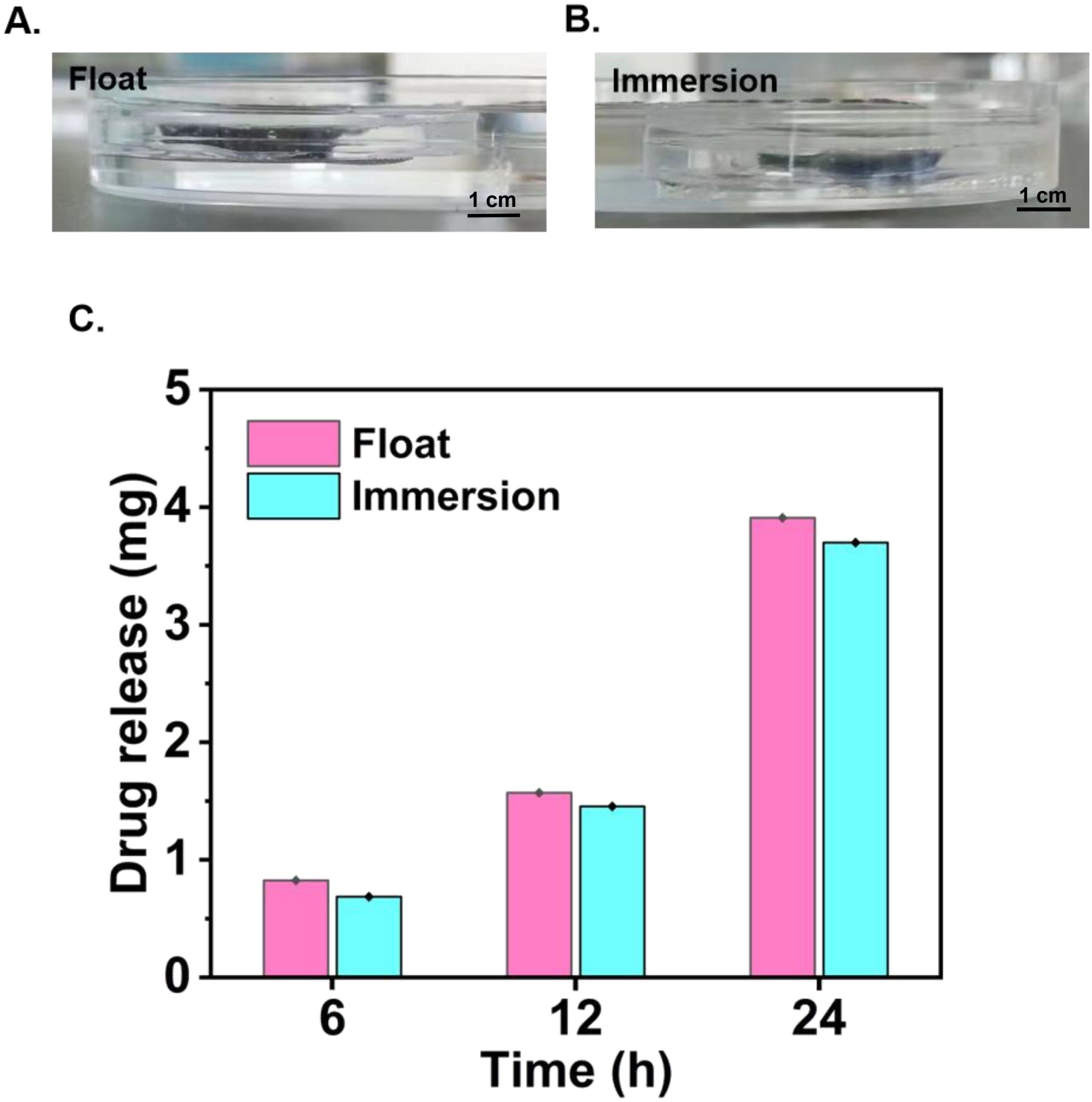

Figure S14. (A) Patch in the floating state. (B) Patch of the immersion state. (C) Amount of drug release when the patch is in the floating and submerged state.

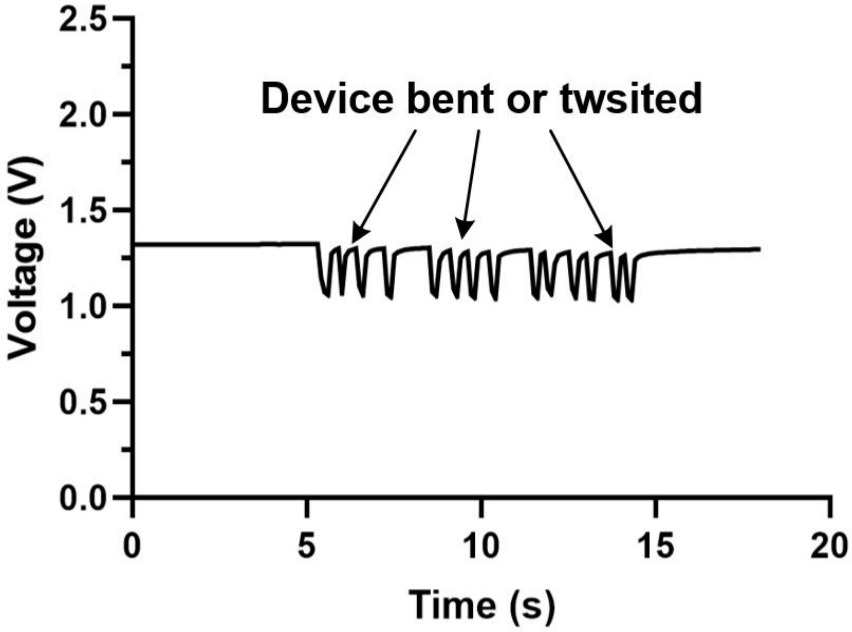

Figure S15. Voltage variation of the device under simulated motion conditions.

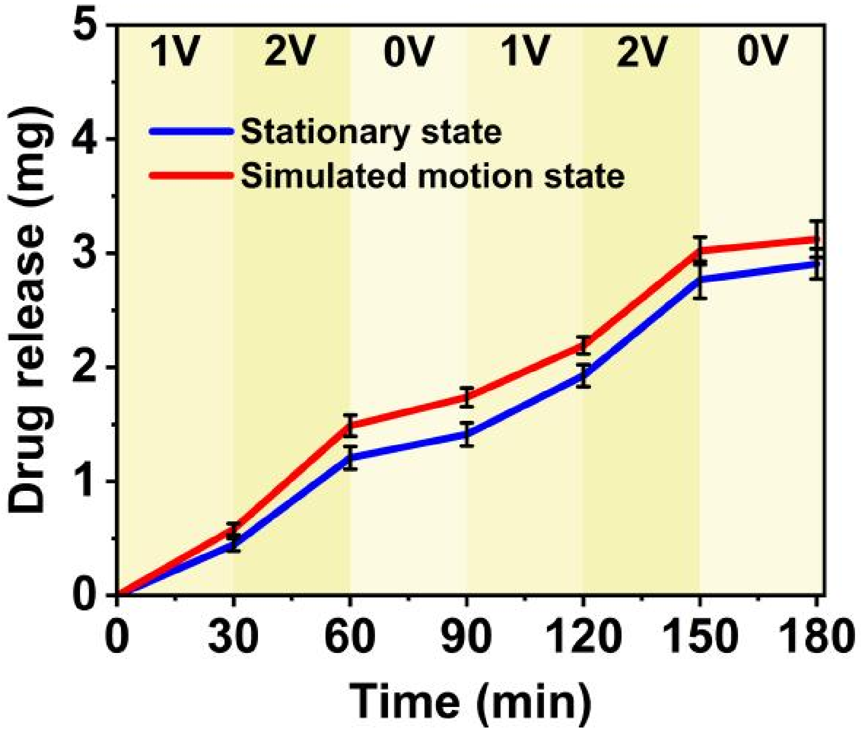

Figure S16. Drug release curves under different motion conditions.

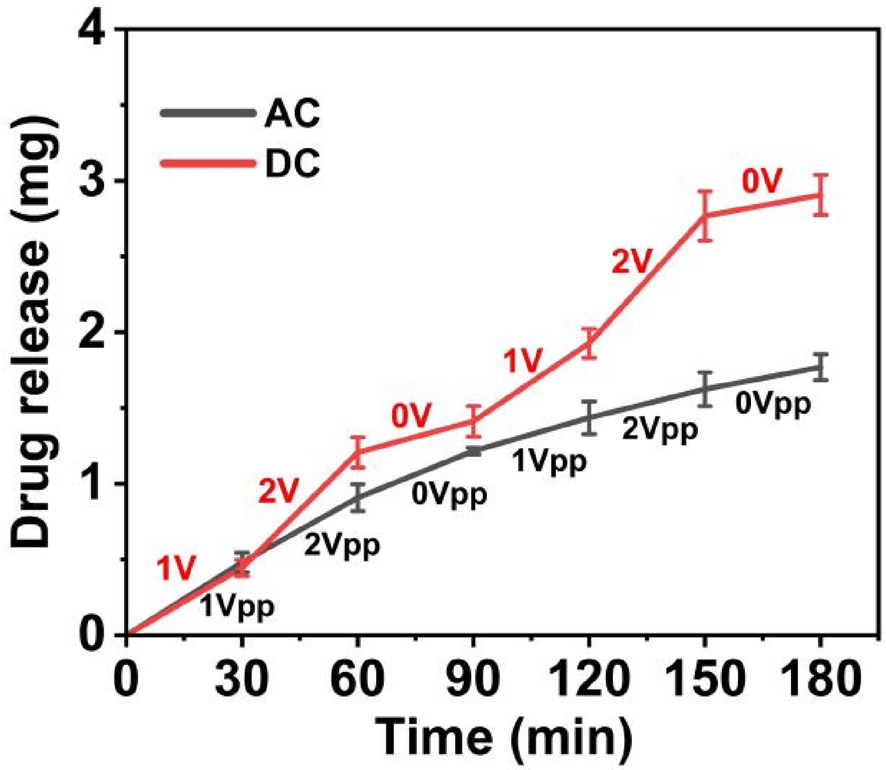

Figure S17. Drug release curves under DC and AC.

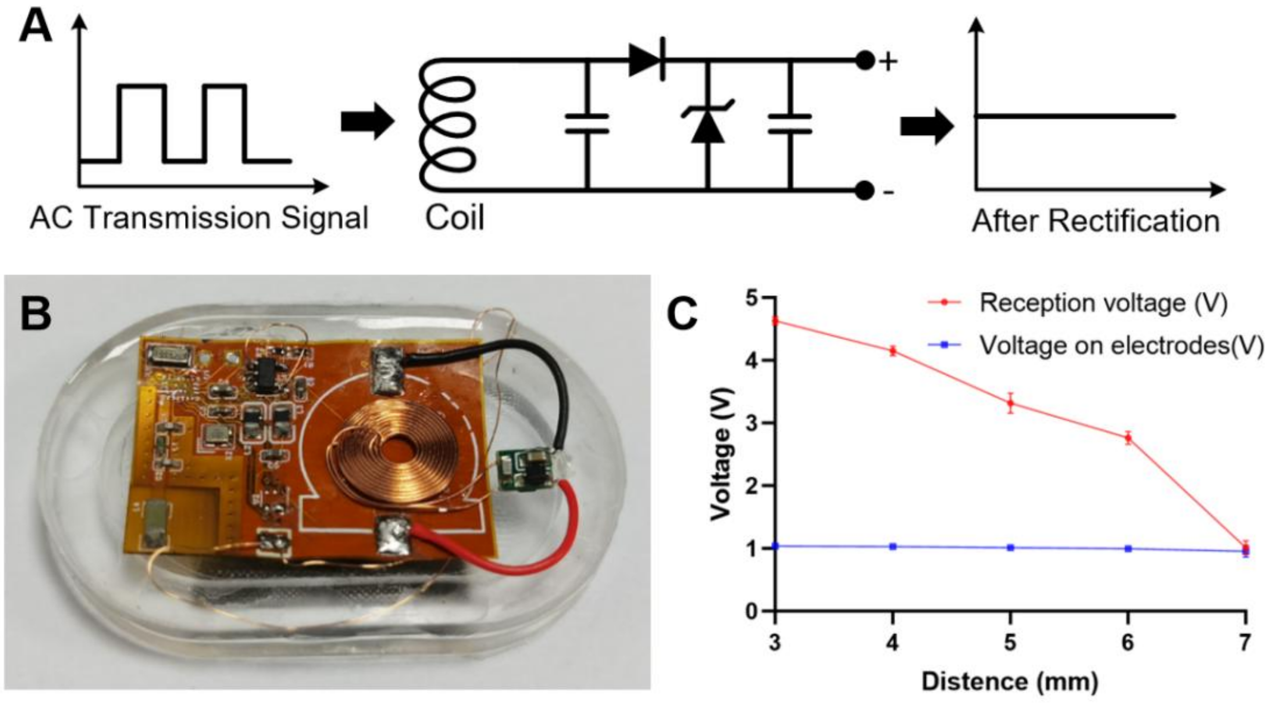

Figure S18. (A) Schematic diagram of wireless charging; (B) Physical image of wireless charging; (C) Experimental results of wireless charging.

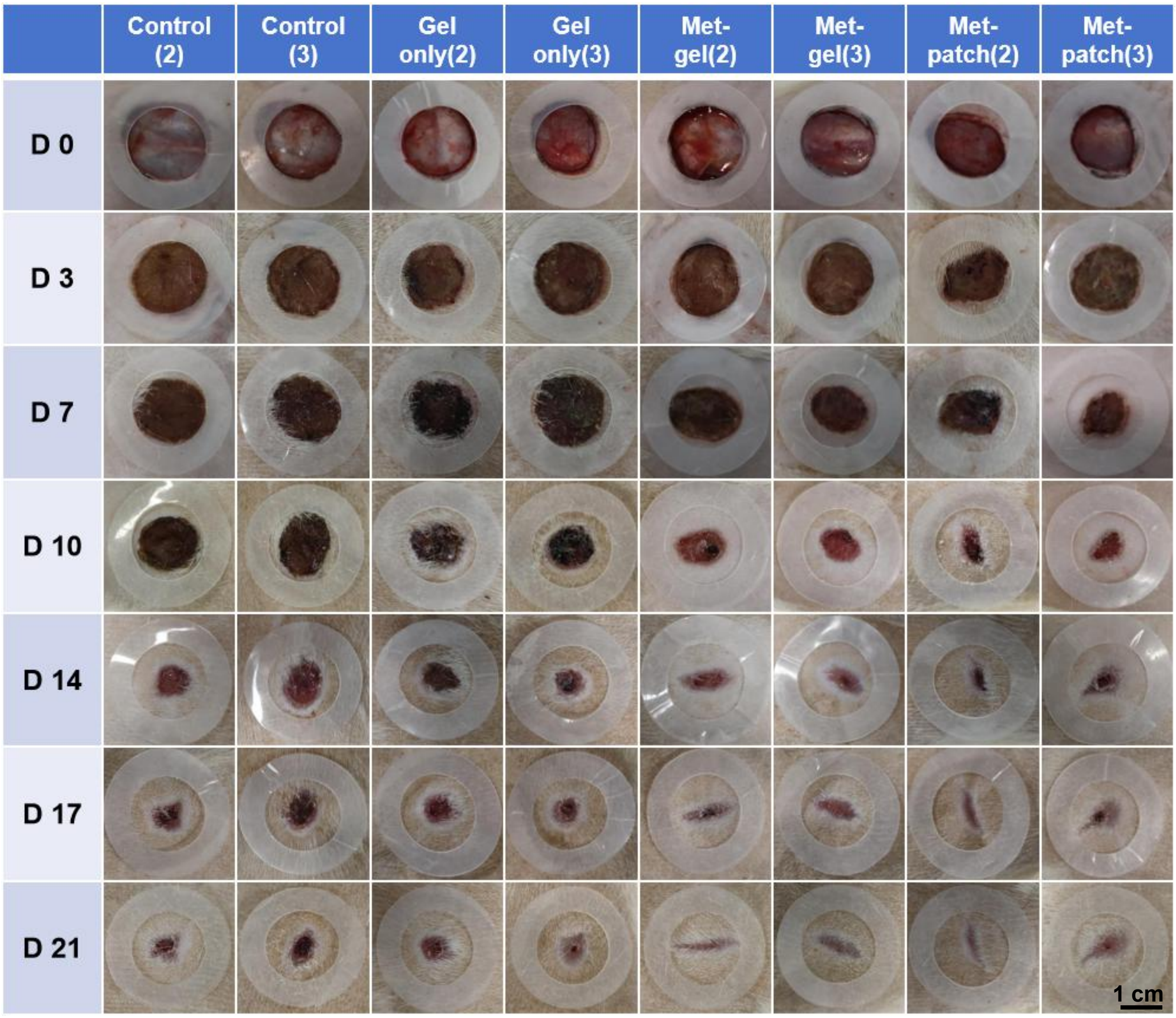

Figure S19. Supplementary data for the animal experiments.

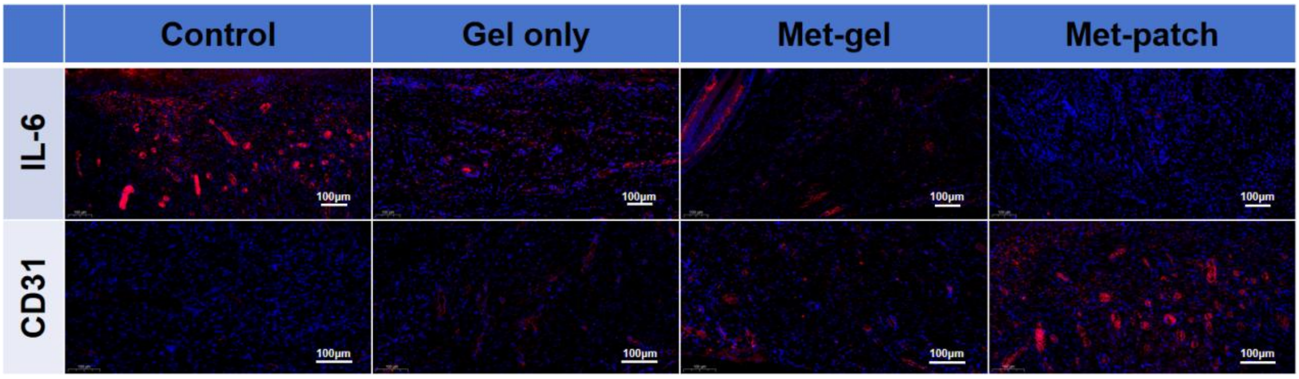

Figure S20. Immunofluorescence staining of IL-6 and CD31 expressed in wound sites

Table S1. Advantages, disadvantages, and drug release efficiency of different stimuli-responsive methods.

Stimulus responsive	Advantages	Disadvantages	Drug release efficiency	References
Electricity	①The switch state can be actively controlled<br>②The cost is relatively low<br>③Can be wirelessly controlled remotely	①Additional equipment	About 20-100%	[1]
Temperature	①Widely applicable<br>②The response speed is fast	①The switch state cannot be actively controlled<br>②Biocompatibility is relatively poor	About 25-95%	[2-3]
pH	①Capable of achieving specific and precise release	①The switch state cannot be actively controlled	About 20-100%	[4-5]
Glucose	①Prolonged release can be achieved<br>②Can be used for the treatment of typical diseases	①The switch state cannot be actively controlled	About 25-100%	[6-7]
Enzyme	①Highly specific release can be achieved	①The switch state cannot be actively controlled<br>②The triggering conditions are relatively stringent	About 50-100%	[8]
Light	①The switch state can be actively controlled<br>②The cost is relatively low<br>③Can be wirelessly controlled remotely	①Difficult to penetrate tissues<br>②It may cause side effects in the human body.	About 10-90%	[9]
Magnetics	①The switch state can be actively controlled<br>②Can be wirelessly controlled remotely	①Magnetic materials are difficult to degrade<br>②The cost is relatively high	About 20-90%	[10]
Redox	①Specific responsive release can be achieved	① The switch state cannot be actively controlled	About 25-95%	[11-12]
Ultrasound	① The switch state can be actively controlled	①Ultrasound may affect the gel structure	About 70%	[13]

References：

[1] J. Qu, X. Zhao, P. X. Ma, B. Guo, *Acta Biomaterialia* **2018**, *72*, 55.
[2] Y. H. Yeo, W. H. Park, *Carbohydrate Polymers* **2021**, *258*, 117705.
[3] W. Kang, J. Liang, T. Liu, H. Long, L. Huang, Q. Shi, J. Zhang, S. Deng, S. Tan, *International Journal of Biological Macromolecules* **2022**, *200*, 99.
[4] A. Celebioglu, A. F. Saporito, T. Uyar, *ACS Sustainable Chemistry & Engineering* **2022**, *10* (14), 4758.

[5] H. Jing, X. Huang, X. Du, L. Mo, C. Ma, H. Wang, *Carbohydrate Polymers* **2022**, *278*, 118993.
[6] R. Yin, K. Wang, S. Du, L. Chen, J. Nie, W. Zhang, *Carbohydrate Polymers* **2014**, *103*, 369.
[7] R. Yin, J. He, M. Bai, C. Huang, K. Wang, H. Zhang, S.-M. Yang, W. Zhang, *Materials Science and Engineering: C* **2019**, *96*, 374.
[8] C.-Y. Wang, M. Sun, Z. Fan, J.-Z. Du, *Chinese Journal of Polymer Science* **2022**, *40* (10), 1154.
[9] C. Liu, P. Yang, J. Li, S. Cao, J. Shi, *Carbohydrate Polymers* **2022**, *295*, 119853.
[10] J. Wu, W. Jiang, R. Tian, Y. Shen, W. Jiang, *Journal of Biomaterials Science, Polymer Edition* **2016**, *27* (15), 1553.
[11] Y. Yang, H. Zhu, J. Wang, Q. Fang, Z. Peng, *ACS Applied Materials & Interfaces* **2018**, *10* (39), 33493.
[12] F. Wang, Q. Zhang, X. Li, K. Huang, W. Shao, D. Yao, C. Huang, *International Journal of Biological Macromolecules* **2019**, *134*, 413.
[13] F.-Z. Yuan, H.-F. Wang, J. Guan, J.-N. Fu, M. Yang, J.-Y. Zhang, Y.-R. Chen, X. Wang, J.-K. Yu, Fabrication of Injectable Chitosan-Chondroitin Sulfate Hydrogel Embedding Kartogenin-Loaded Microspheres as an Ultrasound-Triggered Drug Delivery System for Cartilage Tissue Engineering. In *Pharmaceutics*, **2021**; Vol. 13.
